# Marked decline in forest-dependent small mammals following habitat loss and fragmentation in an Amazonian deforestation frontier

**DOI:** 10.1371/journal.pone.0230209

**Published:** 2020-03-11

**Authors:** Ana Filipa Palmeirim, Manoel Santos-Filho, Carlos A. Peres

**Affiliations:** 1 Laboratório de Vertebrados, Departamento de Ecologia, Universidade Federal do Rio de Janeiro, Rio de Janeiro, Rio de Janeiro, Brazil; 2 School of Environmental Sciences, University of East Anglia, Norwich, United Kingdom; 3 Universidade do Estado de Mato Grosso - UNEMAT, Centro de Pesquisa de Limnologia, Biodiversidade, Etnobiologia do Pantanal, Laboratório de Mastozoologia, Cáceres – Mato Grosso, Brazil; 4 Departamento de Sistemática e Ecologia, Universidade Federal da Paraíba, João Pessoa, Brazil; Museu de Ciències Naturals de Granollers, SPAIN

## Abstract

Agricultural frontier expansion into the Amazon over the last four decades has created million hectares of fragmented forests. While many species undergo local extinctions within remaining forest patches, this may be compensated by native species from neighbouring open-habitat areas potentially invading these patches, particularly as forest habitats become increasingly degraded. Here, we examine the effects of habitat loss, fragmentation and degradation on small mammal assemblages in a southern Amazonian deforestation frontier, while accounting for species-specific degree of forest-dependency. We surveyed small mammals at three continuous forest sites and 19 forest patches of different sizes and degrees of isolation. We further sampled matrix habitats adjacent to forest patches, which allowed us to classify each species according to forest-dependency and generate a community-averaged forest-dependency index for each site. Based on 21,568 trap-nights, we recorded 970 small mammals representing 20 species: 12 forest-dependents, 5 matrix-tolerants and 3 open-habitat specialists. Across the gradient of forest patch size, small mammal assemblages failed to show the typical species-area relationship, but this relationship held true when either species abundance or composition was considered. Species composition was further mediated by community-averaged forest-dependency, so that smaller forest patches were occupied by a lower proportion of forest-dependent rodents and marsupials. Both species richness and abundance increased in less isolated fragments surrounded by structurally simplified matrix habitats (e.g. active or abandoned cattle pastures). While shorter distances between forest patches may favour small mammal abundances, forest area and matrix complexity dictated which species could persist within forest fragments according to their degree of forest-dependency. Small mammal local extinctions in small forest patches within Amazonian deforestation frontiers are therefore likely offset by the incursion of open-habitat species. To preclude the dominance of those species, and consequent losses of native species and associated ecosystem functions, management actions should limit or reduce areas dedicated to pasture, additionally maintaining more structurally complex matrix habitats across fragmented landscapes.

## Introduction

The synergistic effects of habitat loss, fragmentation and degradation have led to a decline in overall species diversity in tropical forests worldwide [1, 2]. Although encompassing both the largest and most biodiverse tropical forest region on Earth, the Amazon has been subject to the highest absolute tropical deforestation rates [3]. In particular, over the last four decades, agricultural frontiers have expanded from neighbouring savannah-like wooded biomes (Cerrado and Caatinga) into the Brazilian Amazon. Such expansion created the so-called Amazonian ‘arc of deforestation’ spanning over 1 million hectares [4], which includes a myriad variable-sized forest patches isolated mostly within cattle pastures and, to a lesser extent, croplands [5].

Typically, species diversity persisting in fragmented landscapes depends on the remaining habitat amount [6, 7], in addition to landscape configuration in terms of habitat area and isolation [8, 9]. Habitat patch area represents a key limiting factor for species population sizes, while the degree of isolation limits species colonization rates [10]. In addition, species diversity is affected by habitat quality of both forest patches and surrounding matrix habitats. Indeed, forest patches are subject to edge effects, which ultimately alter the vegetation structure [11–13] and narrow the spectrum of trophic and structural resources, all of which are aggravated by greater human disturbance including fires, logging and presence of cattle [14]. The surrounding matrix further limits individual dispersal according to varying degrees of matrix hostility, which is often expressed by the structural complexity of vegetation [12, 15].

In fragmented landscapes, local extinctions often result from species that are unable to persist under newly disturbed habitat conditions [16, 17]. However, species composition is additionally susceptible to changes due to the proliferation of common, introduced, habitat generalist and/or open-habitat species [17–19], which can offset extinctions in disturbed habitats [20, 21]. Across the Amazonian deforestation arc, the creation of anthropogenic habitats provides novel opportunities for the expansion of open-habitat species (i.e., those whose geographic distributions are centered in open-habitat biomes) from neighbouring savannah-like biomes. Eventually, species that were already established in non-forest matrix habitats may invade forest remnants, particularly as those become increasingly degraded into poor-quality habitat for forest-dependent species [12].

Amphibian and reptile species typical of open-habitat areas have expanded their distributions into non-native habitats in the Amazon following deforestation [22, 23]. A similar scenario has also been observed for small mammal assemblages, in which species typical of open-habitats occupied not only anthropogenic matrix habitats but also forest fragment edges, suggesting the early stages of a biotic homogenization process [12]. In Neotropical forests, small mammals (rodents and marsupials) are highly diversified in terms of locomotion habits and diet, playing important ecological roles, including seed predation and dispersal [24], pollination [25], and arthropod predation and control [26, 27]. In the aftermath of habitat loss and fragmentation across agricultural frontiers, the loss of small mammal species is potentially compounded by changes in species composition that may entail unprecedented impacts on ecosystem functioning [28].

Here we examine the effects of habitat loss and fragmentation, and any subsequent habitat degradation, on small mammal species persistence in a southern Amazonian deforestation frontier dominated by cattle pastures. Due to relatively fertile and well-drained soils, this region became embedded within the ‘deforestation arc’ agricultural frontier, and succumbed to massive deforestation rates since the late 1970s. Currently, this landscape is comprised of thousands of variable-size forest patches, subjected to different types and degrees of human disturbance [29]. We surveyed small mammals at 19 variable-sized forest patches across a wide range of isolation distances and three continuous forest sites. Additionally, we sampled small mammals in the open-habitat matrix surrounding each forest patch, which allowed us to quantitatively classify each species according to degree of forest-dependency. Across the entire fragmented landscape, we tested the following hypotheses: (1) small mammal diversity–species richness, abundance and composition–is predicted by forest area, in addition to levels of isolation and indicators of habitat quality; (2) changes in small mammal assemblages are mediated by their inherent forest-dependency, if any detrimental effects on forest-dependent species are offset by positive effects on open-habitat species; (3) the relative effect sizes of patch, landscape and habitat-related metrics and species eco-morphological traits predict patterns of species incidence and abundance.

Based on the strong relationship observed between community forest-dependency and forest area, we further predicted changes in community-wide forest-dependency of small mammals across the entire study region since the onset of agricultural frontier expansion before 1985 until the present.

## Material and methods

### Study area

**Fig 1:**
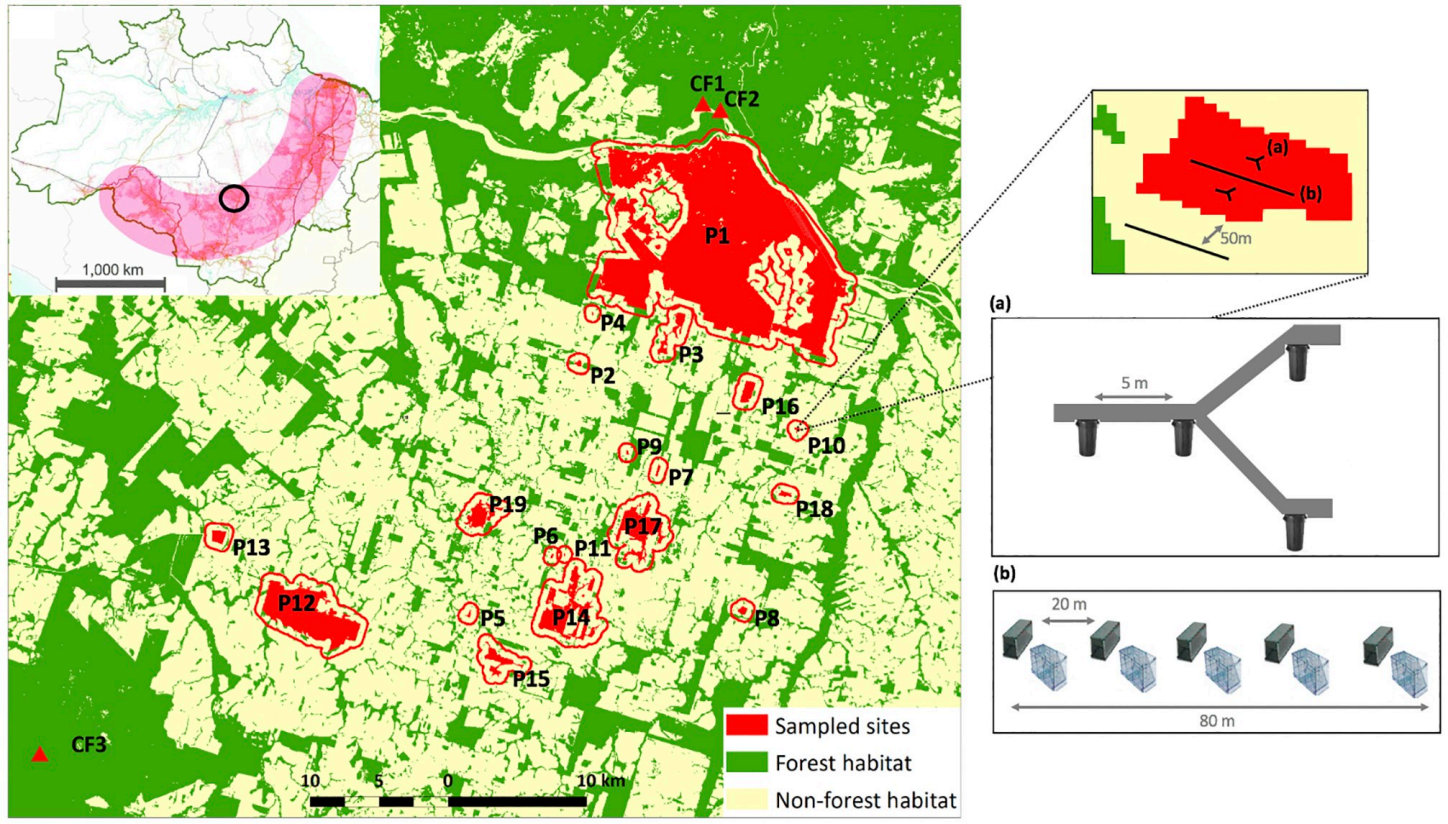
Surveyed sites in Alta Floresta, southern Brazilian Amazon: 19 forest patches (in red and highlighted by a buffer of 1,000 m-radius) and three continuous forest sites (CF1, CF2 and CF3). The inset map shows the location of the study area (black circle) in relation to the ‘deforestation arc’ (pink-coloured) within the Legal Brazilian Amazonia (delimited in green). Surveyed sites are numbered according to S1 Table. The enlarged forest patch (inset) illustrates the sampling design applied to a small patch (< 2 ha): (a) an array of four pitfall-traps; (b) followed by five live-trap stations, each of which including one Sherman and one wire-mesh trap deployed on the forest floor. Distances between traps (stations) are indicated in the figure. See main text for a detailed description of the sampling design.

This study was carried out in the increasingly fragmented landscape of Alta Floresta, State of Mato Grosso, southern Brazilian Amazon (09° 53′ S, 56° 28′ W; Fig 1). The agricultural frontier reached this region in the late 1970s, following a new road which paved the way to the agrarian resettlement of thousands of southern Brazilian farmers [29]. Forests have since been converted into cattle pastures, while remaining forest patches have been subject to logging, burning and selective hunting [29]. By the time sampling was carried out in 2009, the Alta Floresta landscape already included thousands of forest patches of different sizes, shapes and degrees of isolation, which were largely embedded within exotic pastures [30].

Nineteen forest patches of sizes varying across four orders of magnitude (range = 1.4–14,480.5 ha) and degrees of isolation were previously selected within a 50-km radius of the Alta Floresta urban center so a wide range of both patch and landscape scale configurations could be sampled. We maximized the spatial independence between sampling sites by establishing a minimum edge-to-edge distance >1 km. Forest patches were almost or entirely isolated from the continuous forest (S1 Table) and often surrounded by a matrix of managed cattle pastures. As baseline control sites, we additionally selected three continuous primary forest (CF) sites. One CF site was located on the left bank of the Teles Pires River, in the southwestern portion of the landscape, while the other two CF sites, due to the reduced amount of continuous forest remaining in left bank of this River, were located on the right bank (Fig 1).

### Small mammal sampling

Small mammal assemblages were sampled during one trapping session of 10 consecutive nights between February and September in 2009, using both live and pitfall traps. Live trap transects contained five stations spaced by 20-m intervals, each of which including one cage Sherman-like trap (8 × 9 × 23 cm; Gabrisa Ambiental, Cafelândia–SP, Brazil) and one wire-mesh Tomahawk-like trap (14.5 × 14.5 × 41 cm; (Gabrisa Ambiental, Cafelândia–SP, Brazil). Four unbaited pitfall traps (60 L) were also laid out as a Y-shaped sampling unit or array, in which each pitfall bucket was placed at the extremities or at the center of the array. Pitfall traps were 15-m apart from one another and connected by a plastic drift fence 50-cm high and buried 5-cm underground, with 5-m of fence extending beyond the three most extreme pitfalls. During the rainy season, pieces of polystyrene foam were placed inside the buckets to preclude mortality by drowning, and buckets were emptied daily.

Forest patches smaller than 2 ha were sampled by one transect of live-traps and two pitfall-trap arrays, while patches larger than 2 ha and continuous forest sites were sampled by three transects of live traps and four arrays of pitfall traps. Live trap transects were 300-m apart from one another, and each pitfall-trap array intercalated each live-trap transect (see Fig 1). The higher sampling effort on larger forest sites—where overall trap density (S1 Table), and consequently the probability of an individual passing near any given trap, was lower—allowed us to obtain a higher number of individuals therein. In areas adjacent to each forest patch, we additionally installed one live trap transect within the anthropogenic matrix, which was laid out parallel to and 50-m away from the forest edge. All traps were set on the forest floor for comparative purposes between forest patches and their surrounding matrix. Live traps were baited with peanut butter and banana, and both live and pitfall traps were checked early every morning. Sampling effort amount to a total of 21,568 trap-nights across all 22 surveyed sites (15,868 trap-nights in forest patches and CF sites, and 5,700 trap-nights in matrix areas near forest patches).

### Ethics statement

Animals trapping and handling followed the guidelines of the American Society of Mammalogists [31] and was approved by the Instituto Chico Mendes, the appropriate Brazilian government agency (SISBIO license No. 1694–1, request number: 10987) and by the animal care and use committee of the State University of Mato Grosso (‘Universidade do Estado de Mato Grosso’, UNEMAT) [32]. Voucher specimens were euthanized in the field using anaesthetics and preserved in formyl, and subsequently deposited at the Zoological Collection of UNEMAT, in Cáceres, Brazil. Moreover, no specific permissions were required for these study sites and our study does not involve endangered or protected species.

### Patch, landscape and habitat quality variables

Patch and landscape variables were calculated using Landsat imagery for the same year in which sampling was carried out (2009), including patch size and shape, overall forest cover and a forest patch proximity index [33]. For the last two variables, we first considered a set of different buffer area/radii (forest cover: 2.5, 5, 10 and 20 km^2^ buffer; proximity index: 250 m, 500 m and 1,000 m radii). We further selected the most appropriate radial buffer size by performing Generalized Linear Models (GLMs), using species richness, standardized abundance and the first Principal Coordinates Analysis axis (PCoA_1_) of species composition as response variables, while comparing both their Akaike Information Criteria (AIC) [34] and their explanatory power (R^2^). For further analyses, we retained forest cover and the proximity index obtained using the 2.5-km^2^ buffer and 1,000-m radius, respectively (S2 Table). We additionally measured the habitat complexity of the matrix surrounding forest patches, in terms of the degree of vegetation openness. This variable was estimated by direct observation within a 50-m radial area around matrix traps, according to a number of pre-defined classes related to the average pasture and scrub vegetation height (Table 1). Because some patch and landscapes variables could not be obtained for CF sites, we assigned their metrics to closely approximate “real-world” values. Thus, for patch area (Area), we considered an area one order of magnitude larger than our largest forest patch; for island shape (Shape), we considered the area of a circle; for the proximity index (Prox), one order of magnitude greater than our largest forest patch; for forest cover (Cover), the maximum value of 100%; and for matrix complexity (Matrix), the maximum value of 10 (Table 1). Furthermore, since five of all 19 forest patches were somehow connected to other forest remnants (S1 Table), we tested whether lack of complete isolation of those sites induced any bias in our results. To do so, we included this variable (i.e., presence/absence of connectivity to other forest remnants) in a set of preliminary analyses equivalent to those described in the Data Analysis section (see details on preliminary analyses in S3 Table). We did not find evidence for any effect of this additional variable in further explaining our data (S3 Table), so we did not include it in subsequent analyses.

**Table 1:** Description of landscape, patch and habitat quality variables quantified to examine properties of small mammal assemblages in Alta Floresta, southern Brazilian Amazon.

Name (code name)	Variable description	Range (mean ± SD)
*Landscape scale*		
Forest cover (Cover)	Proportion of forest cover within 2.5 km^2^-buffers (%). CF sites = 100%.	5.8–100 (41.2 ± 30.7)
Proximity (Prox)	The sum of all forest patch areas divided by the squared sum of edge-to-edge distances from each focal patch to all patches within a 500 m-buffer; [33]). CF sites = 1.00 x 10^9^.	0.8–5.1 x 10^6^ (4.3 x 10^5^ ± 1.2 x 10^6^)
Matrix complexity (Matrix)	Overall matrix composition: (1) pasture/urban area, (2) pasture/abandoned pasture, (3) abandoned pasture, (4) abandoned pasture/establishment of secondary forest, (5) plantation (e.g. corn)/pasture/ establishment of secondary forest, (6) abandoned pasture/early secondary forest, (7) pasture/plantation/late secondary forest, (8) establishment of secondary forest, (9) early secondary forest, (10) late secondary forest.	1–10 (2.2 ± 2.1)
*Patch scale*		
Patch area (Area)	Total area of each focal forest patch (ha). CF sites = 14,4800 ha.	1.35–14,480 (935.9 ± 3,210)
Patch shape (Shape)	Total perimeter length of each focal forest patch divided by the total patch area. CF sites = 1.00.	1.69–18.88 (5.37 ± 3.89)
*Habitat quality*		
Dominant vegetation (Veg)	Dominant vegetation type within forest fragment/continuous forest according to the categories: (1) low canopy-forest, (2) high-canopy forest with high density of forest-gaps, and (3) high and closed-canopy forest.	1–3 (1.9 ± 0.8)
Fire history (Burn)	Fire history and extent according to the categories: (0) never burned, (1) fire in small area, (2) fire at the forest edges, (3) fire in large area, (4) fire across the whole forest area.	0–4 (2.1 ± 1.2)
Presence of cattle (Cattle)	Presence/absence of domestic cattle incursions within forest patches/continuous forest.	0–1 (0.42 ± 0.49)
Isolation age (Age)	Number of years since the present area. CF2 and 3 = 0 yrs, CF 1 = 1 yr.	1–25 (11.6 ± 8.0)
Logging intensity (Logging)	Information combined from a ranked score of the harvested timber-species profiles, method of extraction, and spatial extent of the timber offtake [30], and grouped according to the categories: (1) none, (2) light, (3) moderate, (4) heavy, (5) very heavy.	1–5 (2.64 ± 1.26)

The range, mean and standard deviation are provided for each variable. Because some patch and landscapes variables could not be obtained for CF sites, we assigned their metrics to closely approximate “real‐world” values which are further indicated.

Habitat quality within forest patches and continuous forest sites included the dominant vegetation type, time (yrs) since isolation, fire history, logging intensity, and presence of bovine cattle. The dominant vegetation type was obtained in the field. The other variables were obtained from interviews with local settlers and, for the fire history and logging intensity, and by additionally quantifying local signs of *in situ* forest fires and cattle tracks (for a detailed description of each variable see Table 1).

### Species traits

We used species geographic range size, body mass, diet and locomotion mode as the main morpho-ecological species traits [35] (each species trait is described in S4 Table, and trait values of individual species are indicated in S5 Table). We also calculated the degree of forest habitat-dependency (FD) for each species, defined as the species-specific ratio between the species abundance (i.e. capture rate) within forest patches and in neighbouring open-habitat matrix areas (log_10_ x + 0.01); using only information obtained from live-traps, as no comparable pitfall-traps were deployed in matrix areas. This allowed us to first classify species within one of two groups: those preferring the matrix, being recorded more often therein and thus classified as ‘open-habitat’ species (FD < 0), and those preferring forest, being recorded more often within forest patches (FD > 0). Secondly, we classified those species preferring forest according to their potential ability to further use the matrix, so that those potentially using the matrix were classified as ‘matrix-tolerant’ (FD > 1.82), while the others likely to completely avoid the matrix were classified as ‘forest-dependent’ (FD < 1.82). The threshold FD = 1.82 corresponds to the maximum value of FD obtained for a species recorded at least once in the matrix. We therefore used this threshold to distinguish species recorded at least once in the matrix (*Euryoryzomys nitidus* and *Proechimys* cf. *roberti*), or species highly abundant within patches further being more likely to use the matrix (*Monodelphis glirina* and *Didelphis marsupialis*), from those recorded exclusively in forest patches. We calculated the standard deviation of each species FD by incorporating standard deviation values of species abundance within forest patches and open-habitat matrix areas into the FD equation. We further obtained the community-averaged FD values by summing the FD values of all individuals recorded at each survey site, and dividing this by the total number of individuals therein. Due to differences in sampling effort per site, species abundances were previously standardized for each site. Community-averaged FD values ranged between 1.61 and 3.04, with higher values corresponding to a higher prevalence of forest-dependent species. We were unable to calculate the forest-dependency index for two species–*Monodelphis kunsi* and *Gracilinanus peruanus*–because they were exclusively detected by pitfall-traps (placed only within forest patches and continuous forest sites). To calculate the community-averaged FD including all species, we assigned FD values from other species for which information was available. For *M*. *kunsi*, we used the FD value obtained for *M*. *glirina*, a closely related species, and for *G*. *peruanus*, we used the FD value obtained for *Caluromys lanatus* since both species were recorded only once throughout the study in a forest patch. Although all captures of *M*. *kunsi* and *G*. *peruanus* amounted to only to 3.81% of all small mammal records (S6 Table), we still considered retaining the approximate values for these species in the community-averaged FD as more informative.

### Data analysis

We first assessed the adequacy of small mammal sampling using the sample coverage estimator [36], which estimates the proportion of the total number of individuals in an assemblage that belongs to the species represented in the sample. Our sample estimates were satisfactory, averaging 0.89 ± 0.15 (0.5–1; S1 Table), so we retained the observed species richness in all subsequent analyses. As in the community-averaged FD calculation, species abundances were previously standardized for each site. Species composition was examined using Principal Coordinates Analysis (PCoA) based on the quantitative Bray–Curtis similarity matrix of species composition.

According to the Habitat Amount Hypothesis (HAH) at the landscape level, the species diversity persisting in fragmented landscapes can be predicted exclusively by the total amount of habitat surrounding sampling sites [7]. In this case, the effects of either patch area or isolation on species diversity provide little, if any, additional explanation beyond the overall habitat amount. To decide whether or not to use forest cover or both patch area and isolation (proximity index) to explain small mammal diversity–species richness, standardized species abundance (log_10_ x) and species composition (PCoA axis 1)–we obtained the percentage of independent effects for each of these metrics (i.e., forest cover, area and proximity index) by applying hierarchical partitioning, using the R package ‘hier.part’ [37]. We found similar support for both the HAH and the central tenets of island biogeography (S7 Table). For comparative purposes with most studies assessing the effects of habitat loss and fragmentation, we therefore retained patch area and isolation in any subsequent analyses.

#### Forest area effects

Considering all 22 sampled sites, we first analysed the effects of forest area (log_10_ x) on each of four response variables—species richness, abundance (log_10_ x), species composition (PCoA_1_) and community-averaged FD—using GLMs with a Gaussian distribution. We decided to analyse the separate effects of forest area because, when considering all 22 sampled sites, patch area and the proximity index were highly correlated (*r* > 0.80), further precluding the inclusion of both variables in the same models. To improve model fitting, we removed three clear outliers from the analyses examining variation in community-averaged FD. When related to forest area, these three patches presented either much higher (patch 9) or much lower (patches 12 and 17) community-averaged FD values, likely because of their unusual forest habitat conditions (see [38]). For example, Patch 17 was characterized by a disproportionally high logging intensity (5.0), compared to the overall values (mean ± SD = 2.64 ± 1.26). After the removal of these outliers, the R^2^ increased from 0.12 to 0.61 when only forest area was considered as an explanatory variable, and from 0.28 to 0.85 when considering the full model including all seven explanatory variables. Thus, these outliers likely corresponded to highly discrepant observations. We further improved data fitting by performing simple GLMs, both including and excluding the quadratic term of the explanatory variable (Area). AIC values were compared between the models, and results are presented for the model showing the lower AIC value [34].

#### Combined effects of habitat, patch and landscape variables

To examine the multi-scale effects of habitat, patch and landscape variables on species diversity, we applied GLMs again considering species richness, species abundance (log_10_ x), species composition (PCoA_1_) and community-averaged FD (excluding outliers: patches 9, 12 and 17), with a Gaussian distribution. Data distribution was evaluated graphically, and a Shapiro test was applied whenever the graphic evaluation was ambivalent. This modeling was restricted to the 19 forest patches surveyed, thereby excluding CF sites. To control for additional high levels of variable inter-dependence, we performed a Pearson correlation matrix, but no variables were observed to be highly correlated (*r* > 0.75). We additionally tested for multicollinearity by calculating the Variance Inflation Factor (VIF) of each independent variable. Patch shape and vegetation complexity were moderately redundant (VIF > 5) [39], and thus excluded from further analyses. We included the quadratic term of Area whenever its inclusion depressed AIC model values [34]. A candidate model set was further constructed using all additive combinations of the seven explanatory variables retained, and models were ranked based on their AICc, using the ‘MuMIn’ R package [40]. To account for model uncertainty in multi-model inference, a model-averaging approach was performed using only the most plausible models (i.e. 0 < ΔAICc > 2, ΔAIC = AIC_i_ − AIC_min_ in which i = i^th^ model). The relative importance (RI) of each variable contained in that model set was obtained by the sum of the Akaike weights of the models in which that variable had been included [41]. Explanatory variables were previously standardized (x = 0, σ = 1) to place coefficient estimates onto the same scale.

#### Species traits vs environmental variables

Considering only forest patches, we examined the relative importance of both species traits and environmental variables in explaining patterns of species incidence (presence-absence) and abundance (log_10_ x), using Generalised Linear Mixed Models (GLMMs) with a logistic and a Gaussian distribution. Due to varying specific-species responses, species identity was considered as the random term. As in previous analyses, we checked for autocorrelation and multicollinearity between variables, but none of the variables were either correlated or multicollinear with any other. We then included seven environmental variables and five species traits–body mass, diet, geographic range, locomotion mode and FD index. Considering all additive combinations of the 12 explanatory variables, we performed model selection and averaging using the same procedures as in the previous analysis. All data analyses were performed in R [42].

#### Multi-year changes in community-wide forest-dependency

As community-averaged FD was strongly predicted by forest area, we were able to extrapolate the community-averaged FD values to all surveyed and unsurveyed forest patches and continuous forest sites occurring in the Alta Floresta region. Using the ArcMap 10.1 [43], we analysed changes in small mammal community-averaged FD on the basis of annual Landsat imagery obtained over 31 years (between 1985 and 2015) for the entire Alta Floresta landscape available from ‘Projeto MapBioma’ [44]. We further applied the observed relationship between the community-averaged FD and forest area to estimate the community-averaged FD for each forest patch and continuous forest sites for 5-year intervals over the entire Landsat chronosequence (1985, 1990, 1995, 2000, 2005, 2010 and 2015). In total, we derived patch-scale estimates for this variable for 155,410 forest patches. The total number of isolated forest patches ranged from 13,542 in 2010 to 33,285 in 1990.

## Results

**Fig 2:**
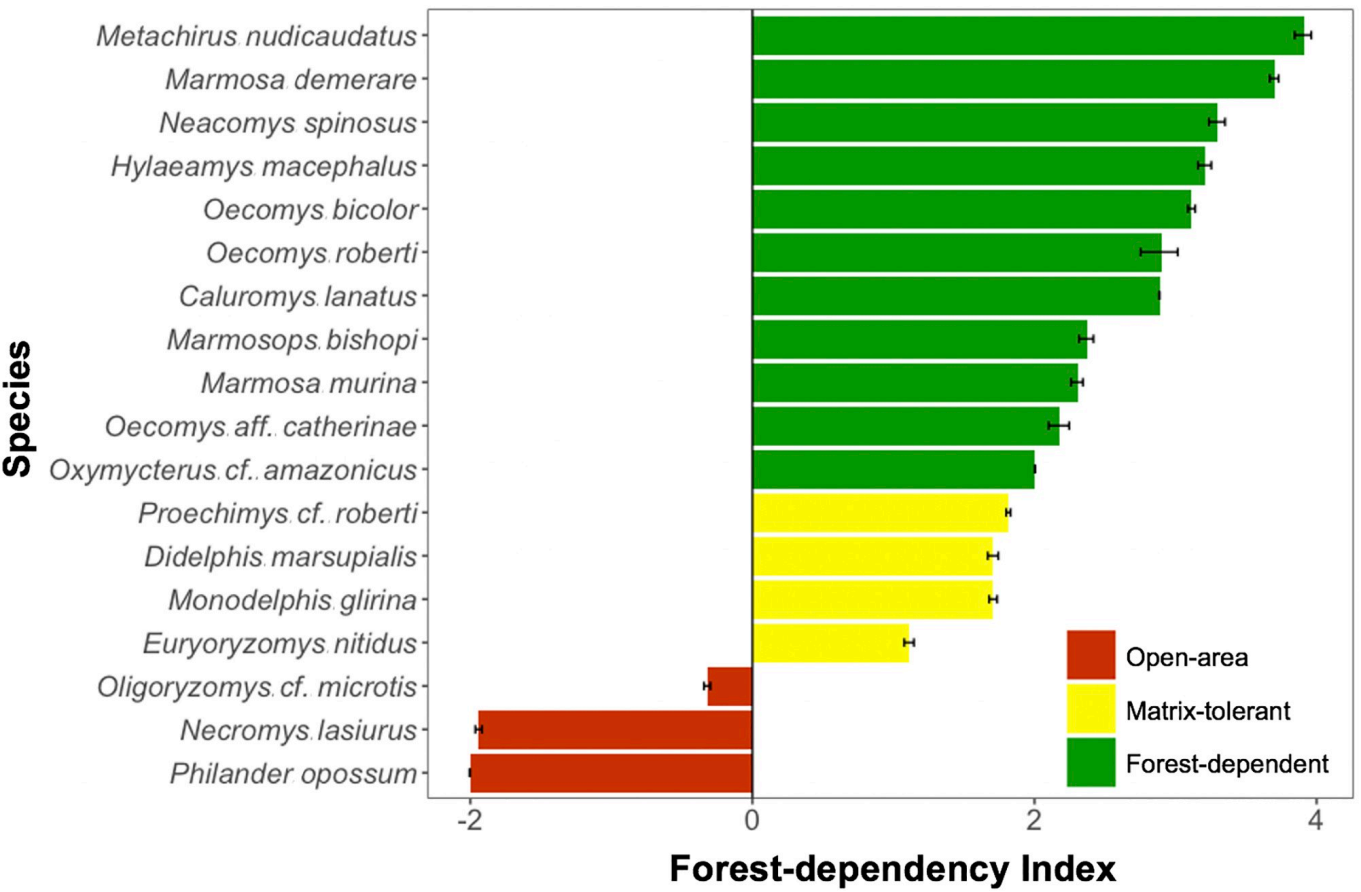
Individual species forest-dependency index (FD) as obtained from the ratio between each species abundance within forest patches and that in neighbouring open-habitat matrix areas (log_10_ x + 0.01). Bars are coloured according to each species classification in terms of forest-dependency: forest-dependent (no individuals were recorded using the matrix; FD > 1.82); matrix-tolerant (at least one individual was recorded using the matrix or species abundance within forest patches was particularly high; 0 < FD < 1.82); and open-area (more individuals were recorded using the matrix than using forest fragments; FD < 0). The threshold FD = 1.82 corresponds to the maximum value of FD obtained for a species recorded at least once in the matrix. FD was obtained using data from live traps only. Prior to analysis, species abundance was standardized according to sampling effort. Error bars correspond to the FD standard error (see main text for details).

A total of 970 small mammals representing 20 species (10 rodents and 10 marsupials) were recorded across all 22 surveyed sites, including matrix habitats surrounding forest patches (S6 Table). This amounted to an overall average of 5.5% and 1.7% of capture success in forest patches/CF sites and matrix areas near forest patches, respectively. The arboreal marsupial *Marmosa demerarae* (*N* = 124) and the terrestrial rodents *Proechimys* cf. *roberti (N* = 126) and *Neacomys spinosus* (*N* = 121) were the most abundant species, while *Caluromys lanatus*, *Gracilinanus peruanus* and *Philander opossum* were recorded only once throughout the sampling (S6 Table). Nineteen species were recorded across forest patches (6.1 ± 2.3 species/patch), 11 species across continuous forest sites (7.7 ± 2.9 species/CF site), and five species across all matrix sites (1.2 ± 0.8 species/matrix site). All five species recorded in the matrix were also recorded in forest patches, except for the marsupial *P*. *opossum*, which was only recorded in the pasture matrix. From all species recorded in the matrix, two were comparatively more often recorded in the matrix than within patches–*Necromys lasiurus* (*N* = 86 ind.) and *Oligorizomys* cf. *microtis* (*N* = 10), and therefore classified as open-habitat species. Two species occasionally used the matrix and were classified as matrix-tolerant, in addition to two other species particularly abundant within forest patches (FD < 1.82; *M*. *glirina* and *D*. *marsupialis*). The remaining 11 species were considered to be strictly forest-dependent (Fig 2).

### Effects of forest area

**Fig 3:**
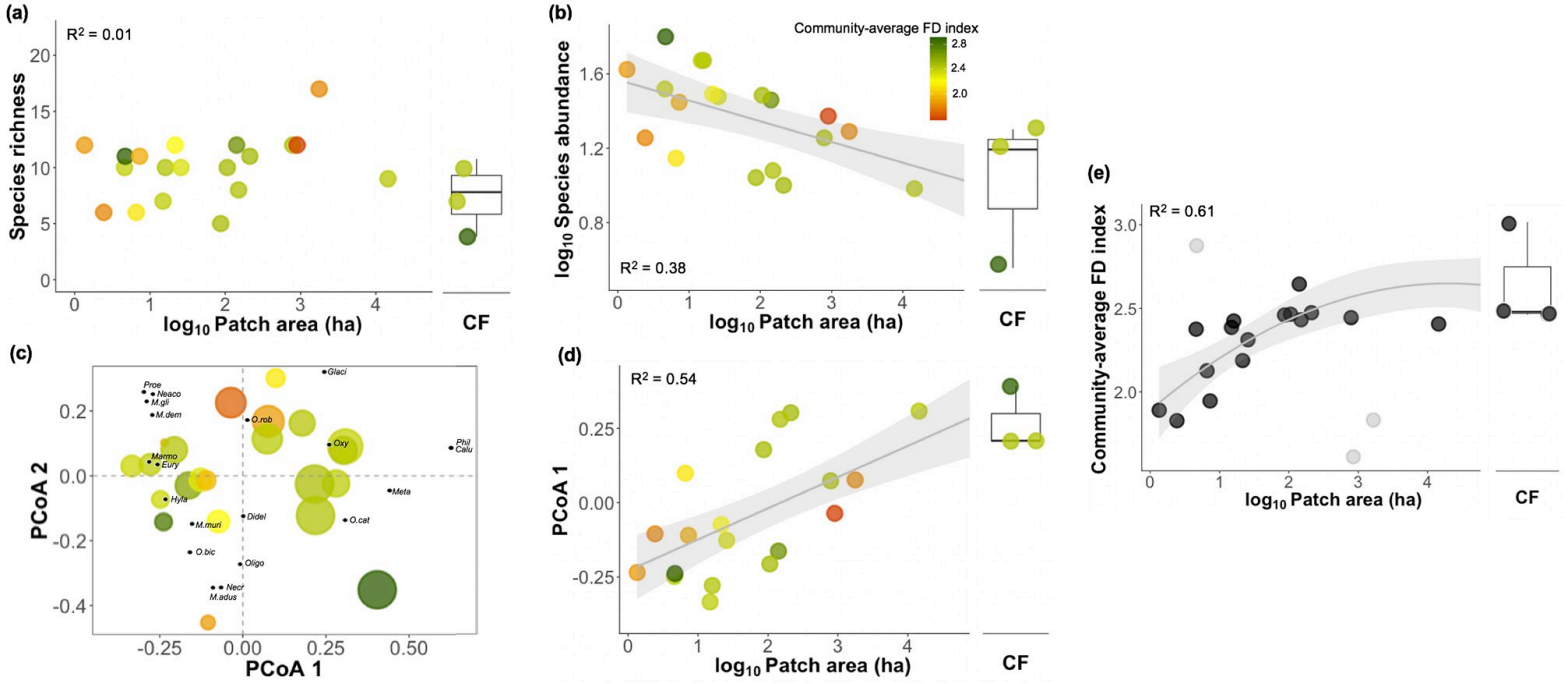
Relationships between species (a) richness and (b) abundance (log_10_ x) and forest area (log_10_); (c) Principal Coordinates Analysis (PCoA) ordination plot; (d) relationship between species composition (PCoA_1_) and (e) community-averaged forest-dependency index (FD) and forest area (log_10_ x). In (a–d), points are colour-coded according to community-average FD values. Lines are the model adjusted for the stronger relationships (*P* ≤ 0.05), and shaded areas represent the 95% confidence region. Grey dots in (e) represent outlier data not included in model fits (patches 9, 12 and 17). Explanation power (R^2^) is indicated for each relationship.

Across all 22 sampled sites, small mammal assemblages did not show the typical positive slope of species-area relationships (Fig 3a). However, forest area was an important predictor of species abundance, which decreased from the continuous forest sites/largest patches to the smallest ones (*β* = –5.397, *P* = 0.005; Fig 3b; S8 Table). Likewise, species composition was affected by forest area (*β* = 0.105, *P* < 0.001; Fig 3d), so that smaller patches tended to harbour more similar sets of species that differed considerably from those at larger forest patches and continuous forest sites (Fig 3c). Such relationships between forest area and both species abundance and composition were mediated by the community-averaged FD index, the variance of which was explained by forest area to a large degree (61%, Fig 3e). The higher community-averaged FD towards smaller fragments (*β*_Area_ = 0.353, *P* = 0.006, *β*Area2 = –0.041, *P* = 0.047; S8 Table) further illustrates the increased fraction of non-forest dependent species (i.e., matrix-tolerant and open-habitat species) therein.

### Combined habitat, patch and landscape effects

**Fig 4:**
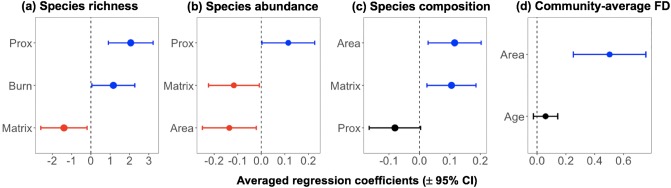
Estimates of averaged models and their 95% confident intervals for predictors of (a) species richness, (b) standardized species abundance (log_10_ x), (c) species composition (denoted by PCoA axis 1), and (d) community-averaged forest-dependency index.

Considering all 19 forest patches, the number of species increased towards less isolated patches (RI_Prox_ = 1.00), previously subject to some degree of burning (RI_Burn_ = 0.70) and surrounded by structurally simpler matrices (e.g. cattle and abandoned pastures; RI_Matrix_ = 1.00, Fig 4a). A similar pattern was observed for relative species abundance which further increased in smaller (RI_Area_ = 1.00) but less isolated forest patches (RI_Prox_ = 0.54), which were also surrounded by less complex matrix areas (RI_Matrix_ = 0.80, Fig 4b). Species composition was strongly predicted by forest patch size (RI_Area_ = 1.00) and matrix complexity (RI_Matrix_ = 1.00; Fig 4c), while the degree of forest-dependency of small mammal assemblages was mainly predicted by forest patch size (RI_Area_ = 1.00, RI_Area_^2^ = 1.00; Fig 4d; S9 Table).

### Species traits vs environmental variables

The relative importance of environmental variables–landscape, patch and habitat quality–was higher than that of species traits in explaining both species incidence and abundance across all forest patches (Table 2). At the landscape scale, species incidence was associated with the proximity index (*β*_Prox_ = 0.570, RI = 1.00), and related to habitat quality, in particular, the severity of burning (*β*_Burn_ = 0.408, RI = 1.00). Species abundance, for instance, was negatively predicted by forest area only (*β*_Area_ = –0.041, *P* < 0.001; Table 2).

**Table 2:** GLMMs explaining overall species abundance (best model) and incidence (average model).

Response variables	Predictors	Estimate	Std. error	*z*-/*t*-value	CI		RI
					2.5	97.5	
*Species abundance*							
Landscape scale	**Area** (log_10_ x)	–0.041	0.012	–3.343	–0.066	–0.017	-
*Species incidence*							
Landscape scale	**Prox** (log_10_ x)	0.570	0.180	3.156	0.216	0.925	1.00
Patch scale	Area	0.081	0.162	0.495	–0.239	0.400	0.10
Habitat quality	**Burn**	0.545	0.175	3.128	0.205	0.895	1.00
	Age	–0.149	0.136	1.093	–0.417	0.118	0.20
	Cattle	0.128	0.152	0.841	–0.1700	0.426	0.12
	Logging	0.064	0.147	0.436	–0.224	0.353	0.05
Species traits	B.mass (log_10_ x)	–0.565	0.414	1.360	–1.379	0.249	0.40
	G.Range	–0.396	0.422	0.934	–1.226	0.435	0.18
	V.Strata	–0.357	0.421	0.846	–1.184	0.470	0.12
	Diet	0.204	0.432	0.471	–0.645	1.053	0.05

For each variable, we indicate the estimate, standard error, *z*-value (for the species incidence model), *t*-value (for the species abundance model), confidence intervals (CI), and relative importance (RI; for the species incidence model). Statistically significant variables are indicated in bold. Habitat variables are described in Table 1; species traits are described in S4 Table and listed for each species in S5 Table, including geographic range in terms of occupied biomes (G.Range), body mass (B.mass; g), diet (Diet), and locomotion mode across vertical forest strata (V.Strata).

### Multi-year changes in community-wide forest-dependency

**Fig 5:**
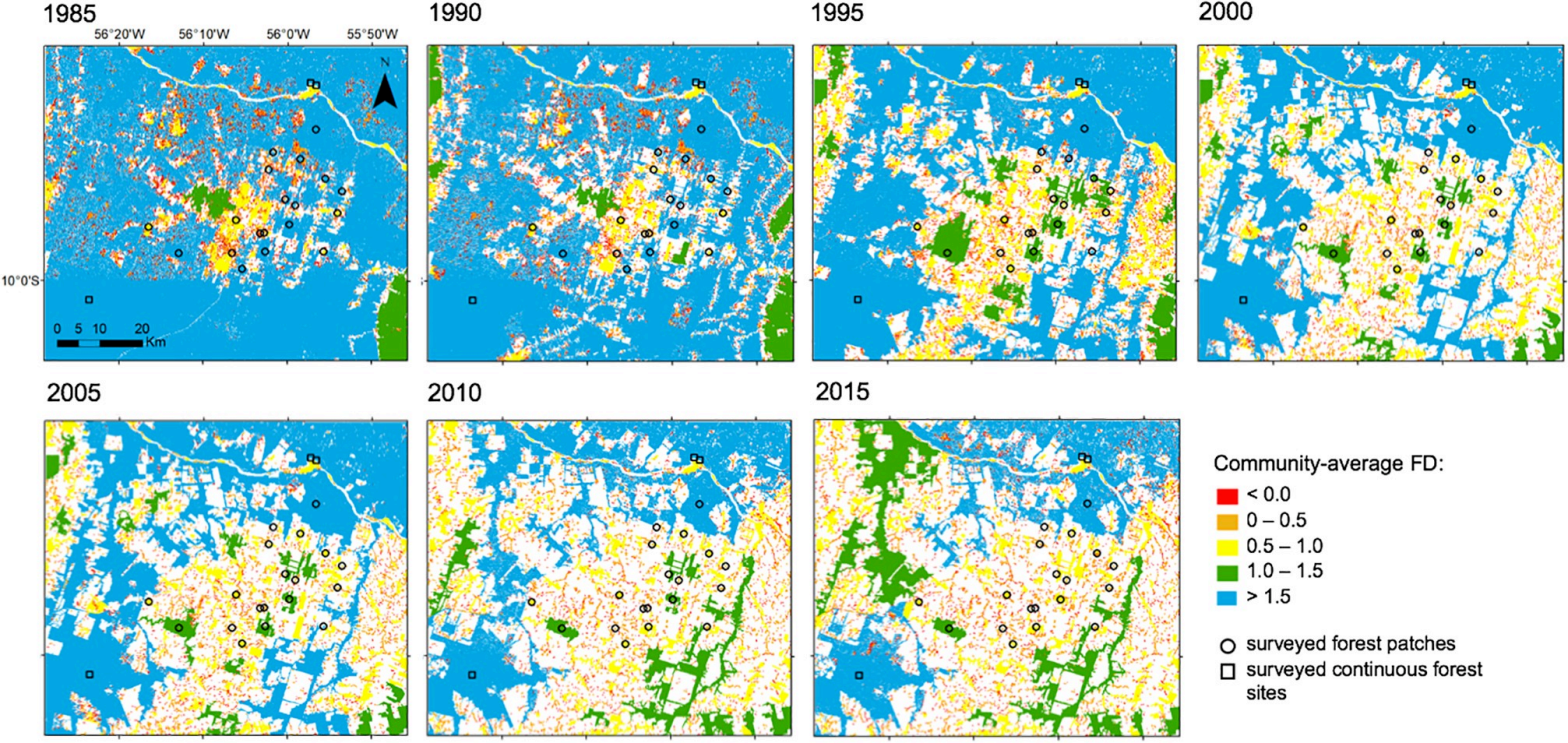
Community-averaged forest-dependency (FD) of small mammal assemblages across the fragmented landscape of Alta Floresta, southern Brazilian Amazon, for 1985, 1990, 1995, 2000, 2005, 2010 and 2015. Forest patches are colour-coded according to the equation: *community-average FD = 0*.*35 log*_*10*_ *forest area– 0*.*04 log*_*10*_ *forest area*^*2*^. Forest area explained 61% of community-averaged FD. For information on species FD values see Fig 2, and on the methods used to estimate community-averaged FD, see main text.

Despite the overall larger amount of forest cover in the early stage of regional scale deforestation in Alta Floresta (1985), a large number of small forest patches (0.06–1.300 ha), for which community-average FD was negative, had been created across the whole region: 30,571 patches (93.3% of all patches). However, this still corresponded to only 1.3% of the entire forest area in the region. In subsequent years, as the agriculture frontier expanded, deforestation resulted in proportionally fewer small forest patches but a larger number of midsize to large patches (Fig 5), which could be colonized by open-habitat species. In recent years, 59.2% of the 1985 forest cover had been lost and forest fragments became increasingly smaller and more isolated from each other (S1 Fig). Deforestation in Alta Floresta therefore culminated in a reverse pattern of forest-dependency of small mammal assemblages, in which non-forest dependent species likely expanded into 75.6% of the entire forested landscape in 2015 (given by the amount of blue-shaded forest in 1985 that was no longer blue in 2015). Across the three decades of deforestation, large forest tracks were relentlessly converted into small to midsized forest patches, many of which rapidly vanishing afterwards. Such changes were followed by severe declines in the overall degree of forest-dependency of small mammal assemblages, which was boosted by the rapid expansion of anthropogenic cattle pastures.

## Discussion

Deforestation frontier expansion across the Alta Floresta region created a heterogeneous patch-matrix mosaic in which local species extinctions were widespread, particularly in non-forest areas and small forest patches. This has been reported for several taxonomic groups, including midsize to large mammals [38, 30], birds [45,46] and trees [47]. In our study, however, we failed to detect any small mammal species-area relationship across the gradient of forest patch size in this landscape (ca. 1 to 14,481 ha). Yet, patch size explained 54% of the species composition which was further mediated by the overall level of community-averaged forest-dependency. As community-wide small mammal forest-dependency decreased in smaller forest sites, local species extinctions were likely to be offset by replacements of non-forest dependent species therein. For example, the small-bodied rodent *N*. *lasiurus*, which we classified as open-habitat species, is known to be a native specialist from the neighbouring *Cerrado* scrub-savannah biome [48]. Our time series of landscape configuration between 1985 and 2015, showing the gradual fragmentation of large tracks of once continuous forest into increasingly smaller forest patches, further illustrate the decline in forest-dependency of small mammal communities in the Alta Floresta region. This overall pattern of non-random ‘functional relaxation’ clearly rewards open-habitat species at the expense of strict forest habitat specialists. Moreover, notwithstanding limitations in terms of sampling methods and season, our small mammal data are consistent with that expected for Alta Floresta, and very similar to those obtained in other Amazonian landscapes (e.g., 19 species [11]; 22 species [49]; 25 species [50]). In fact, although our traps were all placed on the forest floor, pitfall traps are known to be particularly efficient in also recording arboreal and scansorial species [51, 52]. Also, given that we aimed to compare small mammal assemblages in forest remnants of different sizes and continuous forest sites, sampling those largely simultaneously allowed us to maximize comparability.

### Forest area effects

According to either Island Biogeography Theory (IBT) [10] or, alternatively, the Habitat Amount Hypothesis [7], the number of species in fragmented landscapes is expected to increase along the gradient of increasing forest patch size and decreasing isolation, or increasing habitat amount across variable landscape scales, respectively. However, small mammal species richness in Alta Floresta could only be predicted by patch isolation as defined by the proximity index. This suggests that some species were able to traverse the anthropogenic pasture matrix, maintaining high colonization rates in larger and less isolated forest patches (within the 1,000 m-radius threshold from the focal patch), thereby potentially boosting species richness in those patches [10]. This is also the case of small mammal persistence in other fragmented tropical forest landscapes [53, 54], where species continue to occupy small forest patches that are otherwise too small to maintain functionally isolated populations (i.e., when individuals are able to move between patches, also known as the ‘rescue effect’) [55, 56].

In fragmented landscapes, forest patch size is generally more important in explaining species persistence than either isolation [49, 57] or matrix habitat quality [15, 58]. Yet, some limitations of SARs include the fact that in practice some species may also persist in non-forest areas [59]. This appears to be the case in small mammal assemblages of Alta Floresta, where forest area was unrelated to species richness but explained 61% of the community-averaged degree of forest dependency. Colonization of forest patches by open-habitat species can likely offset species extinctions therein [20], further contributing to the lack of a typical species-area relationship in our study landscape. Nevertheless, forest area remained a strong predictor of overall species abundance and composition.

### Matrix complexity and isolation effects

Small mammal species richness was further determined by the degree of complexity of anthropogenic matrix areas surrounding forest patches. Other studies that have similarly failed to detect any species-area relationship also found that matrix habitat quality governs small mammal assemblage structure in forest patches [11, 12, 60]. In general, matrix quality increases with higher structural similarity with adjacent forest patches [15, 61] and high-quality matrix areas are often used by some small mammal species as a secondary habitat [58, 60]. Of the 20 species recorded in Alta Floresta, five (25%) were observed using the matrix, but two of which were most frequently found in forest patches, suggesting that some species may use matrix habitats, but prefer forest areas. Contrary to our expectations, however, the number of small mammal species increased in forest patches surrounded by simplified matrix areas consisting of either active or abandoned cattle pastures, and therefore most structurally divergent from forest patches. Alternatively, these simplified matrix areas might ensure higher abundances of open-habitat specialists [62]. Indeed, the rodent species *N*. *lasiurus* was far more abundant in the matrix than in forest patches. The geographic distribution of this open-habitat species is centered in the *Cerrado* biome of Central Brazil which ranges from natural grasslands to scrubby woodlands [35]. Likewise, in the Amazon-Cerrado contact zone, the open-area rodent *O*. *microtis* is typically found in wet grasslands dominated by shortgrass and sedges [63]. The proportionally higher abundance of open-habitat species in simplified matrix areas likely contribute to their widespread occurrence in forest patches, particularly those that are small, hyper-disturbed, or both. In addition to the overall higher abundance in forest patches surrounded by simplified matrix areas, this hypothesis is further supported by the relationship between species composition in forest patches and the degree of complexity of their surrounding matrix. In a fragmented forest landscape in Central Amazonia, Malcolm [11] similarly observed that matrix assemblages of small mammals were most similar to those in small, edge-dominated forest patches.

Rodents and marsupials were hyper-abundant in small but less isolated forest patches surrounded by active pasture areas, suggesting that species abundance was determined by both within-patch and landscape-scale processes. In other fragmented landscapes, increased small mammal abundance in smaller forest patches compared to larger patches or continuous forest has been widely reported [11, 54–55, 64–65]. Such apparent small mammal overcrowding in small forest patches can be additionally due to the absence of predators [66–67] or reflect a sampling artifact such as higher attractiveness of baited traps given the likely scarcity of alternative food resources [68]. Moreover, the habitat structure of the anthropogenic matrix also modulates species abundances within patches [68, 69]. At the patch scale, small mammals in a landscape 600 km south of Alta Floresta similarly presented species-specific responses to matrix quality that were mediated by habitat preference: forest species benefited from even modest tree cover in the pasture matrix, whereas open-habitat *Cerrado* species benefited from anthropogenic pastures [12]. On one hand, a simplified open-habitat matrix area in Alta Floresta favoured higher abundances of grassland species, resulting in higher number of individuals that eventually occupied adjacent forest patches. On the other hand, lower patch isolation likely favoured inter-patch movements of certain species [70], particularly forest-dependent taxa [49]. As such, poorly isolated patches surrounded by structurally simplified matrix areas contained larger numbers of small mammal species and individuals, while the abundance of open-habitat specialists tended to be particularly high in small forest patches. Small forest patches in Alta Floresta were also characterized by much higher abundances of both birds [71] and mid-sized to larger-bodied mammals [38], which were primarily represented by ubiquitous, habitat-generalist species that could move between neighbouring fragments.

When considered together, environmental variables related to habitat quality (burn intensity) and those measured at the landscape scale (matrix complexity and proximity index) were more important than single eco-morphological species traits in explaining small mammal species incidence and abundance in our study region. These results reflect the importance of environmental variables in determining small mammal assemblages in fragmented landscapes and suggest that considering those variables is still a more efficient strategy to predict changes in small mammal assemblages. Moreover, contrary to our expectations, a history of burn intensity positively affected both species richness and species-specific incidence within forest patches. Fire disturbance is known to negatively affect small mammals [72], although some species can show positive responses [73]. In Alta Floresta, fire disturbance is likely to have created additional habitat conditions within forest sites, further benefiting the incidence of open-habitat species therein.

### Decline in forest-dependency

Small mammal assemblage structure in Alta Floresta changed predictably across the gradient of forest area. Such changes follow the "winner-loser" paradigm [74], in which non-forest dependent species may thrive under conditions of newly hyper-disturbed landscapes, whereas forest-dependent species undergo a spiraling decline. This reflects a much wider biotic homogenization process across many human-dominated tropical forest landscapes. Indeed, overall forest-dependency of small mammal assemblages declined considerably over the past 30 years of deforestation across the Alta Floresta landscape. The cumulative creation of low-quality anthropogenic matrix areas provided ample opportunity for open-habitat species to expand into the Amazon from neighbouring open-habitat biomes. Over our simulated time-series, as remaining forest patches were continuously exposed to further deforestation, with the average area of forest remnants decreasing considerably, open-habitat species gradually gained a strong foothold in forest patches, and now contribute with an increasingly greater proportion of the numerical assemblage in small patches. Overall, according to our simulations, habitat loss and fragmentation across the Alta Floresta region resulted in a reverse pattern of small mammal forest-dependency, which may precipitate important functional changes at the ecosystem level. Many native vertebrate species typical of open-habitat areas have also been observed to expand their distribution into other parts of the Amazon biome [69] and perhaps other tropical forest regions. In several cases, anthropogenic habitat disturbance promoted the expansion of amphibian and reptile species that are better adapted to open-habitat conditions, resulting in the replacement of many strict forest specialists [22, 23]. This non-random process of functional replacement of native biotas deserves far more attention in years to come.

### Conservation implications

Rapid deforestation frontier expansion into the southern Amazon resulted in a novel patch-matrix macro-mosaic, where remaining forest patches are only expected to shrink further [75, 76]. As a consequence, small mammal assemblages in northern Mato Grosso are gradually relinquishing their forest-dependency traits. Despite the lack of any observable small mammal species-area relationship, forest-dependent species will likely continue to undergo local extirpations in small and increasingly disturbed forest patches. Conversely, we expect open-habitat species will continue to thrive both numerically and in spatial extent. Such contrasting species-specific responses should be clearly accounted for in terms of habitat management and countryside planning. In addition, given the structural role of surrounding matrix areas for small mammal population dynamics stranded in forest patches, management actions should also adopt a holistic landscape approach. Currently, only ~40% of the total forest cover persists in this region, which is below the critical 50% threshold at which landscape structure and connectivity are expected to change abruptly [77]. Our results strongly support management efforts that can limit or reduce the aggregate area allocated to agropastoral lands [78], including the protection of sufficiently large forest areas [79], if not more structurally complex interstitial matrix areas throughout the entire fragmented landscape. Moreover, a social perspective considering the human population settled in this area must be further considered in the interest of long-term landscape-scale conservation management. As future conversion of native vegetation will continue to favour open-habitat, habitat-generalist and invasive species across closed-canopy tropical forest landscapes, such management actions are required to preclude the dominance of those species at the expense of native forest-dependent taxa and their associated ecosystem functions.

## Supporting information

S1 Fig Habitat loss in the Alta Floresta region, Southern Brazilian Amazon, between 1985 and 2017. This map was generated based on GIS available by Projeto MapBiomas (2019).
(PDF)

S1 Table Description of each sampling site and trapping effort per site. Information for each forest patch and continuous forest (CF) site used to survey small mammal assemblages in the Alta Floresta region of Southern Amazonia, including geographic coordinates, sampling season, area, connectivity to other remaining fragments, number of traps, trap-nights, trap-density and sample coverage.
(DOCX)

S2 Table Generalized Linear Models performed to select the most appropriate buffer to be used in subsequent analyses measuring proportion of forest cover and proximity index. The buffers considered corresponded to 2.5, 5, 10 and 20 km^2^ for proportion of forest cover, and 500m, 1000m, 1500m for the proximity index.
(DOCX)

S3 Table Summary of Generalized Linear Models (GLMs) performed to examine the effects of connectivity to other landmasses as noted for five forest patches. Akaike Information Criteria (AIC) values denoting the model fitting for each GLM relating small mammal species diversity with landscape, patch and habitat scale variables, both including and excluding the variable ‘connectivity’ (i.e., presence/absence of any connectivity to other forest remnants).
(DOCX)

S4 Table Description of each of the morpho-ecological species traits of small mammal assemblages. Species traits include: geographic range (G.Range), body mass (B.mass; g), Diet, and locomotion mode (V.Strata).
(DOCX)

S5 Table List of morpho-ecological species traits used, in addition to other environmental variables, to understand small mammal occupancy in the fragmented landscape of the Alta Floresta region. Species traits include: geographic range in terms of biomes occupied (G.Range), body mass (B.mass; g), Diet, locomotion mode across the vertical forest strata (V.Strata) and a forest-dependency index (FD).
(DOCX)

S6 Table Number of individuals per species and its overall abundance (%). Information corresponds to the raw number of individuals recorded across 19 forest patches, neighbouring matrix sites and three continuous forest sites.
(DOCX)

S7 Table Proportion of independent effects of forest cover (%), area (log_10_ x) and proximity index (log_10_ x) on species richness (S), overall abundance (Ab, log_10_ x) and species composition (first axis of the Principal Coordinates Analysis) of small mammal assemblages in the Alta Floresta landscape. This analysis aims to examine the strength of the Habitat Amount Hypothesis (HAH) against the Island Biogeography Theory (IBT) applied to fragmented landscapes. HAH was tested considering the proportion of remaining forest within 2.5 km^2^-buffers and IBT with forest area and isolation as indicated by the proximity index considering a buffer with 1000 m-radius.
(DOCX)

S8 Table Generalized Linear Models (GLMs) explaining species richness, standardized species abundance (log_10_ x), species composition (axis 1 of the Principal Coordinates Analysis) and community-averaged forest-dependency (FD) according to forest area (log_10_ x). GLMs were performed including all 19 forest patches and three continuous forest (CF) sites.
(DOCX)

S9 Table Generalized Linear Models (GLMs) explaining species richness, standardized species abundance (log_10_ x), species composition (axis 1 of the Principal Coordinates Analysis) and community-averaged forest-dependency (FD) according to variables related to forest patch, landscape and habitat-quality. GLMs were performed including only forest fragments (N = 19).
(DOCX)

## References

[1] Gibson L, Lee TM, Koh LP, Brook BW, Gardner TA, Barlow J, et al Primary forests are irreplaceable for sustaining tropical biodiversity. Nature 2011; 478:378 10.1038/nature10425 21918513

[2] Newbold T, Hudson LN, Hill SL, Contu S, Gray CL, Scharlemann JP, Borger L, Phillips HR, Sheil D, Lysenko I, Purvis A. Global patterns of terrestrial assemblage turnover within and among land uses. Ecography 2016; 39:1–13.

[3] Peres CA, Gardner TA, Barlow J, Zuanon J, Michalski F, Lees AC, et al Biodiversity conservation in human-modified Amazonian forest landscapes. Biol Conserv 2010; 143:2314–2327.

[4] IPAM. Instituto de Pesquisa Ambiental da Amazônia (Institute of Environmental Research of the Amazon). https://ipam.org.br. Accessed January 2019

[5] Fearnside PM. Deforestation in Brazilian Amazonia: history, rates, and consequences. Conserv Biol 2005; 19:680–688.

[6] Fahrig L. Effects of habitat fragmentation on biodiversity. Annu Rev Ecol Evol 2003; 34:487–515.

[7] Fahrig L. Rethinking patch size and isolation effects: the habitat amount hypothesis. J Biogeogr 2013; 40:1649–1663.

[8] Mendenhall CD, Karp DS, Meyer CF, Hadly EA, Daily GC. Predicting biodiversity change and averting collapse in agricultural landscapes. Nature 2014; 509:213 10.1038/nature13139 24739971

[9] Bueno AS, Peres CA. Patch‐scale biodiversity retention in fragmented landscapes: Reconciling the habitat amount hypothesis with the island biogeography theory. J Biogeogr 2019; 46:621–632.

[10] MacArthur RH, Wilson EO. The theory of island biogeography. Press Princeton, New Jersey; 1967.

[11] Malcolm JR. Edge effects in central Amazonian forest fragments. Ecology 1994; 75:2438–2445.

[12] Santos-Filho M, Peres CA, Da Silva DJ, Sanaiotti TM. Habitat patch and matrix effects on small-mammal persistence in Amazonian forest fragments. Biodivers Conserv 2012; 21:1127–1147.

[13] Benchimol M, Peres CA. Edge-mediated compositional and functional decay of tree assemblages in Amazonian forest islands after 26 years of isolation. J Ecol 2015; 103:408–420.

[14] Barlow J, Lennox GD, Ferreira J, Berenguer E, Lees AC, Mac Nally R, et al Anthropogenic disturbance in tropical forests can double biodiversity loss from deforestation. Nature 2016; 535:144 10.1038/nature18326 27362236

[15] Prevedello JA, Vieira MV. Does the type of matrix matter? A quantitative review of the evidence. Biodivers Conserv 2010; 19:1205–1223.

[16] Devictor V, Julliard R, Jiguet F. Distribution of specialist and generalist species along spatial gradients of habitat disturbance and fragmentation. Oikos 2008; 117:507–514.

[17] Newbold T, Hudson LN, Phillips HRP, Hill SLL, Contu S, Lysenko I, et al A global model of the response of tropical and sub-tropical forest biodiversity to anthropogenic pressures. Proc R Soc B 2014; 281:20141371 10.1098/rspb.2014.1371 25143038 PMC4150326

[18] Filgueiras BK, Tabarelli M, Leal IR, Vaz-de-Mello FZ, Peres CA, Iannuzzi L. Spatial replacement of dung beetles in edge-affected habitats: biotic homogenization or divergence in fragmented tropical forest landscapes? Divers Distrib 2016; 22:400–409.

[19] Lososova Z, Chytry M, Danihelka J, Tichy L, Ricotta C. Biotic homogenization of urban floras by alien species: the role of species turnover and richness differences. J Veg Sci 2016; 27:452–459.

[20] Banks-Leite C, Ewers RM, Metzger JP. Unraveling the drivers of community dissimilarity and species extinction in fragmented landscapes. Ecology, 2012; 93: 2560–2569. 10.1890/11-2054.1 23431587

[21] Kortz AR, Magurran AE. Increases in local richness (α-diversity) following invasion are offset by biotic homogenization in a biodiversity hotspot. Biol Lett 2019; 15:20190133 10.1098/rsbl.2019.0133 31088282 PMC6548741

[22] Gardner TA, Barlow J, Parry LW, Peres CA. Predicting the uncertain future of tropical forest species in a data vacuum. Biotropica 2007; 39:25–30.

[23] Bitar YOC, Juen L, Pinheiro LC, Santos-Costa MCD. Anuran beta diversity in a mosaic anthropogenic landscape in transitional Amazon. J Herpetol 2015; 49:75–82.

[24] Terborgh J, Lopez L, Nuñez P, Rao M, Shahabuddin G, Orihuela G, et al Ecological meltdown in predator-free forest fragments. Science 2001; 294:1923–1926. 10.1126/science.1064397 11729317

[25] Vieira MF, Carvalho-Okano RM, Sazima M. The common opossum (*Didelphis marsupialis*), as a pollinator of *Mabea fistulifera* (Euphorbiaceae). Ciência e Cultura 1991; 43:390–393.

[26] Carvalho FMV, Fernandez FAS, Nessimian JL. Food habits of sympatric opossums coexisting in small Atlantic Forest fragments in Brazil. Mamm Biol 2005; 70:366–375.

[27] Santos-Filho M, Valois SEM, Ignacio ARA, Lazari PR, Chiquito EA, Lázaro WL. Feeding ecology of *Marmosa demerarae* (Thomas, 1905) and *Marmosops bishopi* (Pine, 1981) (Mammalia, Didelphidae) in forest fragments of Southern Amazon. Mastozool Neotrop 2017; 2:409–418.

[28] Lacher TE Jr, Davidson AD, Fleming TH, Gómez-Ruiz EP, McCracken GF, Owen-Smith N, et al The functional roles of mammals in ecosystems. J Mammal 2019; 100:942–964.

[29] Peres CA and Michalski F. Synergistic effects of habitat disturbance and hunting in Amazonian forest fragments In: Emerging Threats to Tropical Forests. University of Chicago Press, Chicago; 2006; pp. 105–127.

[30] Michalski F, Peres CA. Anthropogenic determinants of primate and carnivore local extinctions in a fragmented forest landscape of southern Amazonia. Biol Conserv 2005; 124:383–396.

[31] Sikes RS. Guidelines of the American Society of Mammalogists for the use of wild mammals in research and education. J Mammal 2016; 97:663–688. 10.1093/jmammal/gyw078 29692469 PMC5909806

[32] Comissão de Ética no Uso de Animais da Universidade do Estado de Mato Grosso. http://portal.unemat.br/ceua. Accessed January 2019.

[33] McGarigal K, Cushman SA, Ene E. FRAGSTATS v4: Spatial Pattern Analysis Program for Categorical and Continuous Maps. Computer software program produced by the authors at the University of Massachusetts, Amherst. http://www.umass.edu/landeco/research/fragstats/fragstats.html. Accessed January 2019.

[34] Burnham KP, Anderson DR. Model selection and multi-model inference: a practical information–theoretic approach. Springer-Verlag, London; 2002.

[35] Paglia AP, Fonseca GAB, Rylands AB, Herrmann G, Aguiar LMS, Chiarello AG, et al Lista Anotada dos Mamíferos do Brasil Occasional Papers in Conservation Biology 6 (2nd ed). Arlington: Conservation International; 2012.

[36] Chao A, Jost L. Coverage-based rarefaction and extrapolation: standardizing samples by completeness rather than size. Ecology 2012; 93:2533–2547. 10.1890/11-1952.1 23431585

[37] Walsh C, Mac Nally R, Walsh MC. The hier.part package. Hierarchical Partitioning. 2003; R package version 1.0–2.

[38] Michalski F, Peres CA. Disturbance‐mediated mammal persistence and abundance‐area relationships in Amazonian forest fragments. Conserv Biol 2007; 21:1626–1640. 10.1111/j.1523-1739.2007.00797.x 18173486

[39] Dormann CF, Elith J, Bacher S, Buchmann C, Carl G, Carré G, et al Collinearity: a review of methods to deal with it and a simulation study evaluating their performance. Ecography 2013; 36:27–46.

[40] Bartoń K. MuMIn: multi-model inference. 2016; R package version 1(15):6.

[41] Rhodes JR, McAlpine CA, Zuur AF, Smith GM, Ieno EN. GLMM applied on the spatial distribution of koalas in a fragmented landscape In: Zuur AF, Leno EN, Walker NJ, Saveliev AA, Smith GM, editors. Mixed effects models and extensions in ecology with R. New York: Springer; 2009; p. 469–492.

[42] R Development Core Team. R: A language and environment for statistical computing. R Foundation for Statistical Computing. http://www.R-project.org/. Accessed January 2019.

[43] ESRI. ArcMap 10.1 by Environmental Systems Research Institute, Inc. Redlands; 2012.

[44] Projeto MapBiomas. Coleção v3.1 da Série Anual de Mapas de Cobertura e Uso de Solo do Brasil. http://mapbiomas.org/map#coverage. Accessed January 2019.

[45] Lees AC, Peres CA. Avian life‐history determinants of local extinction risk in a hyper‐fragmented neotropical forest landscape. Anim Conserv 2008; 11:128–137.

[46] Michalski F, Peres CA. Gamebird responses to anthropogenic forest fragmentation and degradation in a southern Amazonian landscape. PeerJ 2017; 5:e3442 10.7717/peerj.3442 28607839 PMC5466001

[47] Michalski F, Nishi I, Peres CA. Disturbance‐mediated drift in tree functional groups in Amazonian forest fragments. Biotropica 2007; 39, 691–701.

[48] Estavillo C, Pardini R, da Rocha PLB. Forest loss and the biodiversity threshold: an evaluation considering species habitat requirements and the use of matrix habitats. PLoS One 2013; 8:e82369 10.1371/journal.pone.0082369 24324776 PMC3853156

[49] Palmeirim AF, Benchimol M, Vieira MV, Peres CA. Small mammal responses to Amazonian forest islands are modulated by their forest dependence. Oecologia 2018; 187:191–204. 10.1007/s00442-018-4114-6 29556713

[50] Santos-Filho M, Da Silva DJ, Sanaiotti TM. Edge effects and landscape matrix use by a small mammal community in fragments of semideciduous submontane forest in Mato Grosso, Brazil. Braz J Biol 2008; 68:703–710. 10.1590/s1519-69842008000400004 19197487

[51] Palmeirim AF, Benchimol M, Peres CA, Vieira MV. Moving forward on the sampling efficiency of neotropical small mammals: insights from pitfall and camera trapping over traditional live trapping. Mammal Res 2019, 64:445–454.

[52] Umetsu F, Naxara L, Pardini R. Evaluating the efficiency of pitfall traps for sampling small mammals in the Neotropics. J Mammal 2006; 87:757–765.

[53] Goodman SM, Rakotondravony D. The effects of forest fragmentation and isolation on insectivorous small mammals (Lipotyphla) on the Central High Plateau of Madagascar. J Zool 2000; 250:193–200.

[54] Pardini R, de Souza SM, Braga-Neto R, Metzger JP. The role of forest structure, fragment size and corridors in maintaining small mammal abundance and diversity in an Atlantic forest landscape. Biol Conserv 2005; 124:253–266.

[55] Laurance WF. Rainforest fragmentation and the structure of small mammal communities in tropical Queensland. Biol Conserv 1994; 69:23–32.

[56] Pires AS, Lira PK, Fernandez FA, Schittini GM, Oliveira LC. Frequency of movements of small mammals among Atlantic Coastal Forest fragments in Brazil. Biol Conserv 2002; 108:229–237.

[57] Watling JI, Donnelly MA. Fragments as islands: a synthesis of faunal responses to habitat patchiness. Conserv Biol 2006; 20:1016–1025. 10.1111/j.1523-1739.2006.00482.x 16922218

[58] Borges-Matos C, Aragón S, da Silva MNF, Fortin MJ, Magnusson WE. Importance of the matrix in determining small-mammal assemblages in an Amazonian forest-savanna mosaic. Biol Cons 2016; 204:417–425.

[59] Pereira HM, Daily GC. Modeling biodiversity dynamics in countryside landscapes. Ecology 2006; 87:1877–1885. 10.1890/0012-9658(2006)87[1877:mbdicl]2.0.co;2 16937624

[60] Umetsu F, Pardini R. Small mammals in a mosaic of forest remnants and anthropogenic habitats—evaluating matrix quality in an Atlantic forest landscape. Landscape Ecol 2007; 22:517–530.

[61] Pfeifer M, Lefebvre V, Peres CA, Banks-Leite C, Wearn OR, Marsh CJ, Butchart SHM, Arroyo-Rodríguez V, Barlow J, Cerezo A, Cisneros L. et al, 2017. Creation of forest edges has a global impact on forest vertebrates. Nature 2017; 551:187–191. 10.1038/nature24457 29088701 PMC5681864

[62] Passamani M, Fernandez FAS. Abundance and richness of small mammals in fragmented Atlantic Forest of Southeastern Brazil. J Nat Hist 2011; 45:553–565.

[63] Lacher TE Jr, Alho CJ. Terrestrial Small Mammal Richness and Habitat Associations in an Amazon Forest–Cerrado Contact Zone 1. Biotropica 2001; 33:171–181.

[64] Glanz WE. Neotropical mammal densities: how unusual is the community on Barro Colorado Island, Panama In: Gentry AH (ed) Four neotropical rainforests. Yale University Press, New Haven, 1990; pp 287–313.

[65] Adler GH, Seamon JO. Distribution of four-eyed opossum *Philander opossum* (Marsupialia, Didelphidae) on small islands In Panama. Mammalia 1996; 60:91–100.

[66] Terborgh J, Lopez L, Tello S. Bird communities in transition: the Lago Guri islands. Ecology 1997; 78:1494–1501.

[67] Lambert TD, Malcolm JR, Zimmerman BL. Amazonian small mammal abundances in relation to habitat structure and resource abundance. J Mammal 2006; 87:766–776.

[68] Laurance WF. Ecological correlates of extinction proneness in Australian tropical rain forest mammals. Conserv Biol 1991; 5:79–89.

[69] Gascon C, Lovejoy TE, Bierregaard Jr, RO, Malcolm JR, Stouffer PC, Vasconcelos HL, et al Matrix habitat and species richness in tropical forest remnants. Biol Conserv 1999; 91:223–229.

[70] Vieira MV, Olifiers N, Delciellos AC, Antunes VZ, Bernardo LR, Grelle CE, Cerqueira R. Land use vs. fragment size and isolation as determinants of small mammal composition and richness in Atlantic Forest remnants. Biol Conserv 2009; 142:1191–1200.

[71] Lees AC, Peres CA. Gap‐crossing movements predict species occupancy in Amazonian forest fragments. Oikos 2009; 118:280–290.

[72] Griffiths AD, Brook BW. Effect of fire on small mammals: a systematic review. Int J Wildland Fire 2014; 23:1034–1043.

[73] Figueiredo MSL, Fernandez FAS. Contrasting effects of fire on populations of two small rodent species in fragments of Atlantic Forest in Brazil. J Trop Ecol 2004; 20:225–228.

[74] McKinney ML, Lockwood JL. Biotic homogenization: a few winners replacing many losers in the next mass extinction. Trends Ecol. Evol. 1999; 14:450–453. 10.1016/s0169-5347(99)01679-1 10511724

[75] Sampaio G, Nobre C, Costa MH, Satyamurty P, Soares‐Filho BS, Cardoso M. Regional climate change over eastern Amazonia caused by pasture and soybean cropland expansion. Geophys Res Lett, 2007; 34.

[76] Nobre CA, Sampaio G, Borma LS, Castilla-Rubio JC, Silva JS, Cardoso M. Land-use and climate change risks in the Amazon and the need of a novel sustainable development paradigm. Proc Natl Acad Sci 2016; 113:10759–10768. 10.1073/pnas.1605516113 27638214 PMC5047175

[77] Michalski F, Peres CA, Lake IR. Deforestation dynamics in a fragmented region of southern Amazonia: evaluation and future scenarios. Environ Conserv 2008; 35:93–103.

[78] Usubiaga-Liaño A, Mace GM, Ekins P. Limits to agricultural land for retaining acceptable levels of local biodiversity. Nat Sustain 2019; 2:491.

[79] Peres CA. Why we need mega-reserves in Amazonian forests. Conserv Biol 2005; 19:728–733.

