# Supplementary figures and images for: Marked decline in forest-dependent small mammals following habitat loss and fragmentation in an Amazonian deforestation frontier
Source: PLoS One. 2020 Mar 11;15(3):e0230209. doi: 10.1371/journal.pone.0230209 (PMC7065764; doi:10.1371/journal.pone.0230209)

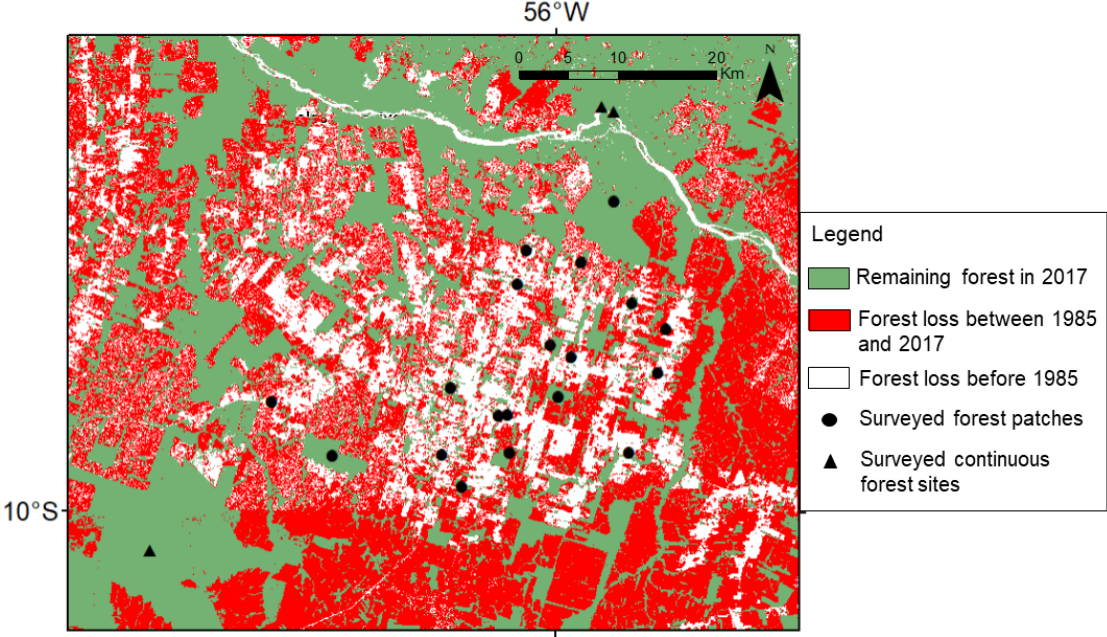

Supplement: S1 Fig — This map was generated based on GIS available by Projeto MapBiomas (2019). (PDF) [file pone.0230209.s001.pdf]
