# Supplementary material for: Marked decline in forest-dependent small mammals following habitat loss and fragmentation in an Amazonian deforestation frontier
Source: PLoS One. 2020 Mar 11;15(3):e0230209. doi: 10.1371/journal.pone.0230209 (PMC7065764; doi:10.1371/journal.pone.0230209)
Supplement: S1 Table — Information for each forest patch and continuous forest (CF) site used to survey small mammal assemblages in the Alta Floresta region of Southern Amazonia, including geographic coordinates, sampling season, area, connectivity to other remaining fragments, number of traps, trap-nights, trap-density and sample coverage. (DOCX) [file pone.0230209.s002.docx]

| **Site name** | **Geographic coordinates** | | | **Sampling season** | **Area (ha)** | **Connectivity** | **Pitfall traps** | | **Live traps** | **Trap-nights** | **Trap-density** | | **Sample coverage** | |  |
| --- | --- | --- | --- | --- | --- | --- | --- | --- | --- | --- | --- | --- | --- | --- | --- |
|  | Lat | Long | |  |  |  |  |  |  |  |  |  |  |  |  |
| Patch 1 | 9°41'22.96"S | | 55°56'27.26"W | Dry | 14,480.5 | 1 | 16 | 90 | | 1060 | | 0.07 | | 0.63 | |
| Patch 2 | 9°46'53.72"S | | 56°02'13.25"W | Wet | 14.6 | 0 | 8 | 30 | | 380 | | 25.59 | | 1.00 | |
| Patch 3 | 9°45'18.46"S | | 55°58'18.30"W | Wet | 141.3 | 1 | 16 | 90 | | 848 | | 6.00 | | 0.95 | |
| Patch 4 | 9°44'56.53"S | | 56°01'43.83"W | Wet | 2.4 | 0 | 8 | 30 | | 380 | | 156.38 | | 1.00 | |
| Patch 5 | 9°56'48.45"S | | 56°06'33.99"W | Dry | 6.6 | 0 | 8 | 30 | | 380 | | 57.84 | | 1.00 | |
| Patch 6 | 9°54'27.89"S | | 56°03'17.55"W | Dry | 4.6 | 0 | 8 | 30 | | 380 | | 82.79 | | 1.00 | |
| Patch 7 | 9°51'06.22"S | | 55°59'06.17"W | Dry | 16.0 | 0 | 8 | 30 | | 380 | | 23.72 | | 1.00 | |
| Patch 8 | 9°56'34.20"S | | 55°55'43.57"W | Dry | 25.7 | 1 | 16 | 60 | | 760 | | 29.53 | | 1.00 | |
| Patch 9 | 9°50'23.64"S | | 56°00'20.18"W | Dry | 4.7 | 0 | 8 | 30 | | 380 | | 81.20 | | 1.00 | |
| Patch 10 | 9°49'27.37"S | | 55°53'33.57"W | Dry | 7.3 | 0 | 8 | 30 | | 380 | | 52.13 | | 1.00 | |
| Patch 11 | 9°54'24.39"S | | 56°02'40.61"W | Dry | 1.4 | 0 | 8 | 30 | | 380 | | 281.48 | | 1.00 | |
| Patch 12 | 9°56'41.87"S | | 56°12'55.50"W | Dry | 1,763.4 | 1 | 16 | 60 | | 760 | | 0.43 | | 0.85 | |
| Patch 13 | 9°53'40.79"S | | 56°16'32.97"W | Dry | 86.9 | 0 | 16 | 60 | | 760 | | 8.75 | | 0.91 | |
| Patch 14 | 9°56'53.55"S | | 56°03'01.52"W | Dry | 787.2 | 0 | 16 | 90 | | 1060 | | 1.35 | | 0.80 | |
| Patch 15 | 9°58'46.30"S | | 56°05'31.03"W | Dry | 211.7 | 1 | 16 | 90 | | 1060 | | 5.01 | | 0.58 | |
| Patch 16 | 9°47'59.99"S | | 55°55'34.78"W | Dry | 106.2 | 0 | 16 | 60 | | 760 | | 7.16 | | 0.93 | |
| Patch 17 | 9°53'17.81"S | | 55°59'44.46"W | Dry | 899.8 | 0 | 16 | 90 | | 1060 | | 1.18 | | 0.90 | |
| Patch 18 | 9°52'01.79"S | | 55°54'02.62"W | Dry | 21.5 | 0 | 8 | 30 | | 380 | | 17.67 | | 1.00 | |
| Patch 19 | 9°52'54.33"S | | 56°06'10.21"W | Dry | 150.0 | 0 | 16 | 90 | | 1060 | | 7.07 | | 0.75 | |
| CF 1 | 10°01'14.04"S | | 56°22'39.33"W | Dry | ∞^a^ | - | 16 | 90 | | 1060 | | 0.01 | | 0.50 | |
| CF 2 | 9°36'30.48"S | | 55°57'21.54"W | Dry | ∞^a^ | - | 24 | 90 | | 1140 | | 0.01 | | 0.85 | |
| CF 3 | 9°36'45.86"S | | 55°56'44.35"W | Dry | ∞^a^ | - | 16 | 90 | | 1060 | | 0.01 | | 0.92 | |

^a^ For analytical purposes, these CF sites were attributed an arbitrary area of 144,800 ha (see Methods) – equivalent to one order of magnitude larger than the largest surveyed patch.

Geographic coordinates (latitude/longitude), season of sampling (dry or wet season), patch size, patch connectivity (i.e., whether the patch as completely isolated [0] or not [1] from nearby land masses), number of pitfalls and live traps deployed, corresponding number of trap-nights, density of traps (number of traps per hectare) and sample coverage estimative are indicated for each survey site. In addition to the data indicated here, a transect of live traps was placed on the ground in the matrix nearby each surveyed site (see the main text for detailed information on sampling design).
