# Supplementary material for: Marked decline in forest-dependent small mammals following habitat loss and fragmentation in an Amazonian deforestation frontier
Source: PLoS One. 2020 Mar 11;15(3):e0230209. doi: 10.1371/journal.pone.0230209 (PMC7065764; doi:10.1371/journal.pone.0230209)
Supplement: S2 Table — The buffers considered corresponded to 2.5, 5, 10 and 20 km2 for proportion of forest cover, and 500m, 1000m, 1500m for the proximity index. (DOCX) [file pone.0230209.s003.docx]

|  | **Response variable** | **Buffer** | **AIC** |
| --- | --- | --- | --- |
| Proportion of forest cover | | | |
|  | S | 2.5 km^2^ | 114.89 |
|  |  | 5 km^2^ | 114.88 |
|  |  | 10 km^2^ | 114.93 |
|  |  | 20 km^2^ | 114.81 |
|  | Ab | 2.5 km^2^ | 3.779 |
|  |  | 5 km^2^ | 4.862 |
|  |  | 10 km^2^ | 4.517 |
|  |  | 20 km^2^ | 4.475 |
|  | PCoA1 | 2.5 km^2^ | –14.164 |
|  |  | 5 km^2^ | –12.326 |
|  |  | 10 km^2^ | –11.669 |
|  |  | 20 km^2^ | –10.932 |
| Proximity Index | |  |  |
|  | S | 500 m | 114.93 |
|  |  | 1000 m | 114.95 |
|  |  | 1500 m | 114.96 |
|  | Ab | 500 m | 9.192 |
|  |  | 1000 m | 8.564 |
|  |  | 1500 m | 8.831 |
|  | PCoA1 | 500 m | –4.503 |
|  |  | 1000 m | –5.31 |
|  |  | 1500 m | –4.962 |

GLMs were performed both the environmental variables and for each of the small mammal diversity measures: species richness (S), species abundance (Ab) and species composition (PCoA axis 1), obtained from the first axis of the PCoA ordination. Values of Akaike Information Criteria are indicated for each model (AIC). See the main text for a detailed description of each environmental variable.
