# Supplementary material for: Marked decline in forest-dependent small mammals following habitat loss and fragmentation in an Amazonian deforestation frontier
Source: PLoS One. 2020 Mar 11;15(3):e0230209. doi: 10.1371/journal.pone.0230209 (PMC7065764; doi:10.1371/journal.pone.0230209)
Supplement: S3 Table — Akaike Information Criteria (AIC) values denoting the model fitting for each GLM relating small mammal species diversity with landscape, patch and habitat scale variables, both including and excluding the variable ‘connectivity’ (i.e., presence/absence of any connectivity to other forest remnants). (DOCX) [file pone.0230209.s004.docx]

| **Response variable** | **Including connectivity** | **Excluding connectivity** |
| --- | --- | --- |
| Species richness | 88.85 | 88.69 |
| Species abundance | 5.63 | 3.73 |
| Species composition | –5.59 | –7.52 |
| Community-averaged FD | –11.16 | –12.59 |

Akaike Information Criteria (AIC) values denoting the model fitting for each GLM relating small mammal species diversity with landscape, patch and habitat quality variables, including and excluding the variable ‘connectivity’ (i.e., presence/absence of any connectivity to other landmasses). GLMs were performed individually for each of the response variables – species richness (number of species), standardized species abundance (log_10_ x), species composition (as denoted by the first axis of the PCoA) and community-average forest-dependency (FD) – considering the full model including the same variables as the models presented in the main text (i.e., forest area, proximity index, matrix complexity, fire history and logging intensity, age of isolation and presence of cattle; details on each variable are described in Table 1 in the main text).
