# Supplementary material for: Marked decline in forest-dependent small mammals following habitat loss and fragmentation in an Amazonian deforestation frontier
Source: PLoS One. 2020 Mar 11;15(3):e0230209. doi: 10.1371/journal.pone.0230209 (PMC7065764; doi:10.1371/journal.pone.0230209)
Supplement: S4 Table — Species traits include: geographic range (G.Range), body mass (B.mass; g), Diet, and locomotion mode (V.Strata). (DOCX) [file pone.0230209.s005.docx]

| **Species trait** | **Description** | **Variable type** |
| --- | --- | --- |
| Geographic range (G.Range) | Number of biomes occupied by each species. | Categoric |
| Body mass (G.Range) | Maximum body mass (g). | Continuous |
| Diet (Diet) | Energy consumed as measured from the average in their dietary classification: frugivorous [1], granivorous/seed predator [2], insectivorous [3], omnivorous [4]. For example, for species classified as frugivorous/granivorous = (1 + 2)/2 = 1.5. | Continuous |
| Locomotion habitat (V.Strata) | Vertical forest strata mostly used: semi-fossorial [1], terrestrial [2], scansorial [3] and arboreal [4]. | Categoric |

Description of each of the small mammal species traits.
