# Supplementary material for: Marked decline in forest-dependent small mammals following habitat loss and fragmentation in an Amazonian deforestation frontier
Source: PLoS One. 2020 Mar 11;15(3):e0230209. doi: 10.1371/journal.pone.0230209 (PMC7065764; doi:10.1371/journal.pone.0230209)
Supplement: S5 Table — Species traits include: geographic range in terms of biomes occupied (G.Range), body mass (B.mass; g), Diet, locomotion mode across the vertical forest strata (V.Strata) and a forest-dependency index (FD). (DOCX) [file pone.0230209.s006.docx]

| **Species** | | **G.Range^a^** | **B.mass (g)** | **Diet^b^** | **Locomotion** | |
| --- | --- | --- | --- | --- | --- | --- |
| Marsupials | |  |  |  |  | |
|  | *Caluromys lanatus* | Am, AF, Ce, Pt | 350–520 | Fr/On | Arboreal | |
|  | *Didelphis marsupialis* | Am | 1000–1700 | Fr/On | Scansorial | |
|  | *Gracilinanus peruanus^c^* | Ce, Ca, Pt | 13–40 | In/On | Arboreal | |
|  | *Marmosa murina* | Am, AF, Ce, Pt | 52 | In/On | Scansorial | |
|  | *Marmosa demerarae* | Am, AF, Ce, Ca | 90–150 | In/On | Arboreal | |
|  | *Marmosops bishopi* | Am | 17–22 | In/On | Scansorial | |
|  | *Metachirus nudicaudatus* | Am, AF, Ce, Pt | 300–480 | In/On | Terrestrial | |
|  | *Monodelphi glirina* | Am | 50 | In/On | Terrestrial | |
|  | *Monodelphis kunsi* | Ce | 20 | In/On | Terrestrial | |
|  | *Philander opossum* | Am, Ce, Pt | 280–700 | In/On | Scansorial | |
| Rodents | |  |  |  |  | |
|  | *Euryoryzomys nitidus* | Am | 55–70 | Fr/Gr | Terrestrial | |
|  | *Hylaeamys megacephalus* | Am, AF, Ce, Pt | 60 | Fr/Gr | Terrestrial | |
|  | *Neacomys spinosus* | Am, Ce | 31 | In/On | Terrestrial | |
|  | *Necromys lasiurus* | Am, AF, Ce, Ca, Pt, Pp | 40–80 | Fr/On | Terrestrial | |
|  | *Oecomys aff. catherinae* | AF, Ce, Ca | 70 | Fr/Se | Arboreal | |
|  | *Oecomys bicolor* | Am, Ce, Pt | 28 | Fr/Se | Arboreal | |
|  | *Oecomys roberti* | Am, Ce, Pt | 240 | Fr/Se | Arboreal | |
|  | *Oligoryzomys* cf. *microtis* | Am | 20 | Fr/Gr | Scansorial | |
|  | *Oxymycterus* cf. *amazonicus* | Am | 76 | In/On | Semi-fossorial | |
|  | *Proechimys* cf. *roberti* | Am, Ce | 191 | Fr/Gr | Terrestrial | |
| ^a^Amazon (Am), Atlantic forest (AF), Brazilian savannah like Cerrado (Ce), Caatinga (Ca), Pantanal (Pt)  ^b^ Fr – frugivorous, Se – seed predator, Gr – granivorous, In – insectivorous, On - omnivorous  ^c^ This species has been recently separated from *G*. *agilis* and no information on specific traits was found, so that we used that information available for *G. agilis* which is indeed phylogenetically, and thus ecologically, very similar. | | | | | |  |
