# Supplementary material for: Marked decline in forest-dependent small mammals following habitat loss and fragmentation in an Amazonian deforestation frontier
Source: PLoS One. 2020 Mar 11;15(3):e0230209. doi: 10.1371/journal.pone.0230209 (PMC7065764; doi:10.1371/journal.pone.0230209)
Supplement: S6 Table — Information corresponds to the raw number of individuals recorded across 19 forest patches, neighbouring matrix sites and three continuous forest sites. (DOCX) [file pone.0230209.s007.docx]

| **Species** | **No. of individuals** | | | **Overall abundance (%)** |
| --- | --- | --- | --- | --- |
|  | **Patch** | **Matrix** | **CF** |  |
| Marsupials |  |  |  |  |
| *Caluromys lanatus* | 1 |  |  | 0.10 |
| *Didelphis marsupialis* | 18 |  |  | 1.86 |
| *Glacilinanus peruanus* | 1 |  |  | 0.10 |
| *Marmosa murina* | 28 |  | 11 | 4.02 |
| *Marmosa demerarae* | 108 |  | 16 | 12.78 |
| *Marmosops bishopi* | 76 |  | 26 | 10.52 |
| *Metachirus nudicaudatus* | 6 |  |  | 0.62 |
| *Monodelphis glirina* | 97 |  | 1 | 10.10 |
| *Monodelphis kunsi* | 21 |  | 15 | 3.71 |
| *Philander opossum* |  | 1 |  | 0.10 |
| Rodents |  |  |  | 0.00 |
| *Euryoryzomys nitidus* | 48 | 2 | 8 | 5.98 |
| *Hylaeamys megacephalus* | 36 |  | 20 | 5.77 |
| *Neacomys spinosus* | 113 |  | 8 | 12.47 |
| *Necromys lasiurus* | 21 | 85 | 1 | 11.03 |
| *Oecomys* aff*. catherinae* | 4 |  | 3 | 0.72 |
| *Oecomys bicolor* | 29 |  |  | 2.99 |
| *Oecomys roberti* | 9 |  | 3 | 1.24 |
| *Oligoryzomys* cf. *microtis* | 17 | 9 |  | 2.68 |
| *Oxymycterus amazonicus* | 2 |  |  | 0.21 |
| *Proechimys* cf. cf. *roberti* | 123 | 1 | 2 | 12.99 |
| Total | 758 | 98 | 114 |  |
