# Supplementary material for: Marked decline in forest-dependent small mammals following habitat loss and fragmentation in an Amazonian deforestation frontier
Source: PLoS One. 2020 Mar 11;15(3):e0230209. doi: 10.1371/journal.pone.0230209 (PMC7065764; doi:10.1371/journal.pone.0230209)
Supplement: S7 Table — This analysis aims to examine the strength of the Habitat Amount Hypothesis (HAH) against the Island Biogeography Theory (IBT) applied to fragmented landscapes. HAH was tested considering the proportion of remaining forest within 2.5 km2-buffers and IBT with forest area and isolation as indicated by the proximity index considering a buffer with 1000 m-radius. (DOCX) [file pone.0230209.s008.docx]

| **Response variable** | **% Forest cover** | **Forest area (log_10_ x)** | **Proximity Index (log_10_ x)** |
| --- | --- | --- | --- |
| S | 49.89 | 46.00 | 4.1 |
| Ab (log10 x) | 32.21 | 50.58 | 17.20 |
| PCoA1 | 30.17 | 50.94 | 18.89 |

This analysis aimed to test the Habitat Amount Hypothesis (HAH) over an adaptation of the Island Biogeography Theory (IBT) to fragmented landscapes. HAH was tested considering the proportion of remaining forest within 2.5 km^2^-buffer and IBT with forest area and isolation as given by the proximity index considering a buffer with 1,000 m-radius.
