# Supplementary material for: Marked decline in forest-dependent small mammals following habitat loss and fragmentation in an Amazonian deforestation frontier
Source: PLoS One. 2020 Mar 11;15(3):e0230209. doi: 10.1371/journal.pone.0230209 (PMC7065764; doi:10.1371/journal.pone.0230209)
Supplement: S8 Table — GLMs were performed including all 19 forest patches and three continuous forest (CF) sites. (DOCX) [file pone.0230209.s009.docx]

| **Response variable** | | **Estimate** | | | | **Std. error** | ***t*-value** | | | ***P*-value** | |
| --- | --- | --- | --- | --- | --- | --- | --- | --- | --- | --- | --- |
| *Species richness* | |  | | | |  |  | | |  | |
|  | Intercept | | 9.898 | | 1.075 | | | 9.207 | <0.001 | |  |
|  | Area | | –0.099 | | 0.403 | | | –0.245 | 0.809 | |  |
| *Species abundance* | |  | | | |  |  | | |  | |
|  | Intercept | 37.038 | | | | 4.548 | 8.145 | | | <0.001 | |
|  | Area | –5.397 | | | | 1.705 | –3.166 | | | 0.005 | |
| *Species composition (PCoA1)* | | | |  | | | |  |  | |  |
|  | Intercept | –0.229 | | | | 0.058 | –3.979 | | | 0.001 | |
|  | Area | 0.105 | | | | 0.022 | 4.864 | | | <0.001 | |
| *Community-average FD index* | | | |  | | | |  |  | |  |
|  | Intercept | 1.890 | | | | 0.120 | 15.706 | | | <0.001 | |
|  | Area | 0.353 | | | | 0.111 | 3.164 | | | 0.006 | |
|  | Area^2^ | –0.041 | | | | 0.019 | –2.147 | | | 0.047 | |

For each model, we indicate estimate, standard error, *t*- and *P*-values. CF sites were assigned with an arbitrary area of 14,4800 ha, equivalent to one order of magnitude larger than the largest surveyed fragment. The GLM regarding the community-average FD index included 16 forest patches and three CF sites, due to the prior exclusion of three outliers (patches 9, 12 and 17).
