# Supplementary material for: Marked decline in forest-dependent small mammals following habitat loss and fragmentation in an Amazonian deforestation frontier
Source: PLoS One. 2020 Mar 11;15(3):e0230209. doi: 10.1371/journal.pone.0230209 (PMC7065764; doi:10.1371/journal.pone.0230209)
Supplement: S9 Table — GLMs were performed including only forest fragments (N = 19). (DOCX) [file pone.0230209.s010.docx]

| **Response variables** |  |  | |  | | **Parameters** | | | | |  | | | | |  |
| --- | --- | --- | --- | --- | --- | --- | --- | --- | --- | --- | --- | --- | --- | --- | --- | --- |
|  | Intercept | | Age | | Burn | | Area | Area^2^ | Prox | Matrix | | **K** | **AICc** | **ΔAICc** | **ω_i_** | |
| **Species richness** | 10.000 | |  | | 1.162 | |  | - | 2.163 | –1.272 | | 4 | 87.7 | 0.00 | 0.260 | |
|  | 10.000 | |  | |  | |  | - | 1.837 | –1.678 | | 3 | 89.4 | 1.66 | 0.133 | |
| (null m.) | 10.000 | |  | |  | |  | - |  |  | | 1 | 95.2 | 7.47 | 0.006 | |
| RI |  | |  | | 0.70 | |  |  | 1.00 | 1.00 | |  |  |  |  | |
| **Species abundance** | 1.371 | |  | |  | | –0.151 | - | 0.115 | –0.127 | | 4 | –2.5 | 0.00 | 0.208 | |
|  | 1.371 | |  | |  | | –0.100 | - |  | –0.093 | | 3 | –1.0 | 1.48 | 0.099 | |
|  | 1.371 | |  | |  | | –0.139 | - |  |  | | 2 | –0.5 | 1.95 | 0.078 | |
| (null m.) | 1.371 | |  | |  | |  | - |  |  | | 1 | 4.0 | 6.51 | 0.008 | |
| RI |  | |  | |  | | 1.00 |  | 0.54 | 0.80 | |  |  |  |  | |
| **Species composition** | –0.044 | |  | |  | | 0.130 | - | –0.081 | 0.115 | | 4 | –13.2 | 0.00 | 0.270 | |
|  | –0.044 | |  | |  | | 0.093 | - |  | 0.091 | | 3 | –12.4 | 0.87 | 0.175 | |
| (null m.) | –0.044 | |  | |  | |  | - |  |  | | 1 | –2.5 | 10.76 | 0.001 | |
| RI |  | |  | |  | | 1.00 |  | 0.61 | 1.00 | |  |  |  |  | |
| **Community-average FD** | 2.300 | |  | |  | | 0.525 | –0.388 |  |  | | 3 | –10.0 | 0.00 | 0.265 | |
|  | 2.300 | | 0.059 | |  | | 0.457 | –0.303 |  |  | | 4 | –8.5 | 1.50 | 0.125 | |
| (null m.) | 2.300 | |  | |  | |  |  |  |  | | 1 | >3.1 | >13.10 | <0.001 | |
| RI |  | | 0.32 | |  | | 1.00 | 1.00 |  |  | |  |  |  |  | |

Variables are coded as: years since isolation (Age), burning intensity (Burn), patch size (Area and Area^2^ – to represent the quadratic term of this variable), Proximity Index (Prox) and matrix complexity (Matrix). Area^2^ was included only in the GLM regarding the community-average FD index. We provide the set of most plausible models, i.e., Akaike weights (W*i*) > 0.001, which are ordered by Akaike information criterion for small samples values (AICc), in addition to the null model. K = number of parameters included in each model; ΔAICc = AICc*_i_* – AICc*_min_*, *i* = *i^th^* model; RI = relative importance of the averaged model. The variables related to the presence of cattle and logging intensity are not indicated since those were not retained in the set of most plausible models. The GLM regarding the community-average FD index included 16 forest patches and three CF sites, due to the prior exclusion of three outliers (patches 12, 17 and 9).
